# Comparative genomics reveals low levels of inter- and intraspecies diversity in the causal agents of dwarf and common bunt of wheat and hint at conspecificity of *Tilletia caries* and *T. laevis*

**DOI:** 10.1186/s43008-022-00098-y

**Published:** 2022-06-07

**Authors:** Somayyeh Sedaghatjoo, Bagdevi Mishra, Monika K. Forster, Yvonne Becker, Jens Keilwagen, Berta Killermann, Marco Thines, Petr Karlovsky, Wolfgang Maier

**Affiliations:** 1 Institute for Epidemiology and Pathogen Diagnostics, Julius Kühn Institute (JKI) - Federal Research Centre for Cultivated Plants, Messeweg 11-12, 38104 Brunswick, Germany; 2 Senckenberg Biodiversity and Climate Research Centre, Senckenberganlage 25, 60325 Frankfurt am Main, Germany; 3 Bavarian State Research Center for Agriculture, Institute for Crop Science and Plant Breeding, Vöttinger Straße 38, 85354 Freising, Germany; 4 Institute for Biosafety in Plant Biotechnology, Julius Kühn Institute (JKI) - Federal Research Centre for Cultivated Plants, Erwin-Baur-Str. 27, 06484 Quedlinburg, Germany; 5 Faculty of Biological Sciences, Institute of Ecology, Evolution and Diversity, Goethe University, Max-von-Laue-Str. 13, 60438 Frankfurt am Main, Germany; 6 Molecular Phytopathology and Mycotoxin Research, University of Goettingen, Grisebachstrasse 6, 37077 Goettingen, Germany

**Keywords:** Fungal pan-genomes, Functional genomics, Closely related fungal species, Trimethylamine biosynthesis, Basidiomycota

## Abstract

*Tilletia caries* and *T. laevis*, which are the causal agents of common bunt, as well as *T. controversa,* which causes dwarf bunt of wheat, threaten especially organic wheat farming. The three closely related fungal species differ in their teliospore morphology and partially in their physiology and infection biology. The gene content as well as intraspecies variation in these species and the genetic basis of their separation is unknown. We sequenced the genome of four *T. caries*, five *T. controversa*, and two *T. laevis* and extended this dataset with five publicly available ones. The genomes of the three species displayed microsynteny with up to 94.3% pairwise aligned regions excluding repetitive regions. The majority of functionally characterized genes involved in pathogenicity, life cycle, and infection of corn smut, *Ustilago maydis,* were found to be absent or poorly conserved in the draft genomes and the biosynthetic pathway for trimethylamine in *Tilletia* spp. could be different from bacteria. Overall, 75% of the identified protein-coding genes comprising 84% of the total predicted carbohydrate utilizing enzymes, 72.5% putatively secreted proteins, and 47.4% of effector-like proteins were conserved and shared across all 16 isolates. We predicted nine highly identical secondary metabolite biosynthesis gene clusters comprising in total 62 genes in all species and none were species-specific. Less than 0.1% of the protein-coding genes were species-specific and their function remained mostly unknown. *Tilletia controversa* had the highest intraspecies genetic variation, followed by *T. caries* and the lowest in *T. laevis.* Although the genomes of the three species are very similar, employing 241 single copy genes *T. controversa* was phylogenetically distinct from *T. caries* and *T. laevis*, however these two could not be resolved as individual monophyletic groups. This was in line with the genome-wide number of single nucleotide polymorphisms and small insertions and deletions. Despite the conspicuously different teliospore ornamentation of *T. caries* and *T. laevis*, a high degree of genomic identity and scarcity of species-specific genes indicate that the two species could be conspecific.

**Supplementary Information:**

The online version contains supplementary material available at 10.1186/s43008-022-00098-y.

## Introduction

The basidiomycete genus *Tilletia* (Tilletiales, Exobasidiomycetes, Ustilaginomycotina) comprises about 186 described species causing smut disease on *Poaceae* (Vánky 2012; Denchev and Denchev 2013, 2018a, b; Li et al. 2014; Denchev et al. 2018). *Tilletia* species are biotrophs that do not develop specialized cellular infection structures, but form so-called local interaction zones in the host tissue (Begerow et al. 2014). The term bunt is used for cereal-infecting species of *Tilletia* that produce teliospores in the ovary of the host plant (Carris et al. 2006). The infection of cereal crops by bunt species remains asymptomatic up to culm elongation (Purdy et al. 1963). The infected seeds smell like fish due to the production of trimethylamine (Hanna et al. 1932; Nielsen 1963). Contaminated seeds are not suitable for human and animal consumption at a certain infection level and must be treated accordingly for use as seeds in organic and conventional farming.

Three kinds of bunt diseases are known from wheat species (*Triticum* spp.) namely common, dwarf, and karnal bunt. Only common and dwarf bunt affect wheat production in Central Europe, where they are under phytosanitary regulation for seed certification in organic and conventional farming. *Tilletia caries* (syn*. T. tritici*) and *T. laevis* (syn*. T. foetida*) cause common bunt of wheat (Woolma and Humphrey 1924; Vánky 2012), a disease that occurs in wheat-growing areas worldwide (Hoffmann 1982; Goates 1996). Dwarf bunt is caused by *T. controversa,* which is reported to be limited to higher elevations (Goates 1996) or regions with prolonged cooler temperatures (Carris 2010). However, in recent years the disease has also been observed to extend to lowland regions in Germany (Rudloff et al. 2020). *Tilletia controversa* is economically important for international seed trading because it is a quarantine pathogen in several countries (Mathre 1996; Whitaker et al. 2001; Peterson et al. 2009; Jia et al. 2013).

*Tilletia caries*, *T. controversa*, and *T. laevis* differ in several biological and physiological features. Firstly, the morphology of teliospores varies from smooth in *T. laevis* to deep and broadened reticulations in *T. controversa* and an intermediate form in *T. caries*. Secondly, the teliospores of *T. caries* and *T. laevis* germinate within a week at 12 to 15 °C under illumination or in dark, while germination of *T. controversa* teliospores requires up to eight weeks at the optimum temperature of 3 to 5 °C and light is essential for germination (Purdy et al. 1963). Furthermore, the infection of wheat by common bunt pathogens occurs before the emergence of the coleoptile, whereas *T. controversa* attacks the same organ after emergence (Carris 2010). Disease symptoms differ moderately between common and dwarf bunt. Substantial wheat stunting and enhanced tillering occur in dwarf bunt and its severity varies among wheat cultivars (Goates 1996; Carris 2010), while stunting in common bunt diseased wheat is not readily distinguishable. Despite the different morphological and physiological features, molecular phylogenetic analysis based on three loci could not resolve the three species unequivocally (Carris et al. 2007). However, a phylogenomic study of five *Tilletia* species based on 4,896 single-copy orthologous genes but limited specimens sampling (one *T. caries*, two *T. controversa*, two *T. laevis*, three *T. indica*, and two *T. walkeri*) suggested that they are distinct (Nguyen et al. 2019).

Despite the growing concern about common and dwarf bunt as major threat to especially organic wheat production due to a limited number of durably resistant cultivars (Ruzgas and Liatukas 2008; Matanguihan et al. 2011; Aydoğdu and Kaya 2020), and the fact that *T. controversa* is a quarantine pathogen, little is known about the genomic structure and gene contents of the three species.

Here, we report draft genome sequences of four *T. caries* isolates, five *T. controversa* isolates, and two isolates of *T. laevis,* obtained from single teliospore cultures that except for one isolate of *T. laevis*, originated from recent European populations. The sequenced isolates were selected from a sample collection of common and dwarf bunt characterized by morphological characters and germination behavior of teliospores and later by MALDI-TOF MS (Forster et al. 2022). The genome sequences of these 11 isolates were analyzed together with five additional publicly available common and dwarf bunt genomes (Nguyen et al. 2019) to provide a first insight into the genomic features as well as genomic diversity within and between these three important pathogens.

## Methods

### Isolates and single teliospore cultures

Isolates of four *T. caries*, five *T. controversa*, and two *T. laevis* were whole-genome sequenced in this study (Table 1). To obtain DNA for genome sequencing, cultures of *T. caries*, *T. controversa,* and *T. laevis* were grown from single teliospores. For the production of single teliospore cultures, teliospores were surface-sterilized as described by Castlebury et al. (2005). Briefly, bunt balls were crushed using a pair of sterile fine-point forceps and the wheat ovary tissue was carefully removed. The teliospores were immersed in 0.26% (v/v) NaClO (Carl Roth, Karlsruhe, Germany) for 30 s, pelleted by centrifugation for 10 s and rinsed twice with sterile, distilled water. For germination, surface-sterilized teliospores were streaked on 1.5% water-agar and incubated either at 5 °C under constant light (*T. controversa* germination) or at 15 °C in darkness (*T. caries* and *T. laevis* germination). A single germinated teliospore of each specimen was then transferred to M-19 agar medium (Trione 1964) using a sterile needle. Fungal cultures on M-19 were maintained at 15 °C in the dark to establish colonies of mononucleate, non-pathogenic, and non teliosporogenic hyphal stage for nucleic acid extraction. The medium was supplemented with penicillin G (240 mg/L) and streptomycin sulfate (200 mg/L). The mycelium was freeze-dried (Christ ALPHA1-4 LSC, Martin Christ Gefriertrocknungsanlagen, Osterode am Harz, Germany) at −40 °C for 48 h and kept at 4 °C until use. Duplicates of the single teliospore cultures obtained in this study were deposited at the CBS-KNAW culture collection of the Westerdijk Fungal Biodiversity Institute (Utrecht, The Netherlands) under the accession numbers provided in Table 1.

**Table 1:** List of the *Tilletia* isolates used for genome comparison

Taxon	Isolate (Accession number^a^)	Collection year	Geographical origin	Host	BioProject accession	Sequencing technology	References
*T. caries*	AA11 (CBS 144825)	2015	Austria	*Triticum aestivum*	PRJEB40624	Illumina HiSeq 4000	This study
*T. caries*	AI (CBS 145171)	2015	Italy	*Triticum durum*	PRJEB40624	Illumina HiSeq 4000	This study
*T. caries*	AO (CBS 145172)	2014	Germany	*Triticum aestivum*	PRJEB40624	Illumina HiSeq 4000	This study
*T. caries*	AZH3 (CBS 145166)	2015	Switzerland	*Triticum aestivum*	PRJEB40624	Illumina HiSeq 4000	This study
*T. caries*	DAOMC 238032	1996	USA	*Triticum* sp.	PRJNA317434	Illumina HiSeq	Nguyen et al. (2019)
*T. controversa*	DAOMC 236426	1998	Canada	*Triticum* sp.	PRJNA317433	Illumina HiSeq, MiSeq, PacBio	Nguyen et al. (2019)
*T. controversa*	DAOMC 238052	1997	Canada	*Triticum* sp.	PRJNA393324	Illumina MiSeq	Nguyen et al. (2019)
*T. controversa*	OA2 (CBS 145169)	2015	Austria	*Triticum aestivum*	PRJEB40624	Illumina HiSeq 4000	This study
*T. controversa*	OL14 (CBS 145167)	2014	Germany	*Triticum aestivum*	PRJEB40624	Illumina HiSeq 4000	This study
*T. controversa*	OR (CBS 144827)	2013	Germany	*Triticum aestivum*	PRJEB40624	Illumina HiSeq 4000, PacBio RS II	This study
*T. controversa*	OV (CBS 145170)	2011	Germany	*Triticum aestivum*	PRJEB40624	Illumina HiSeq 4000	This study
*T. controversa*	OW (CBS 145168)	2013	Germany	*Triticum aestivum*	PRJEB40624	Illumina HiSeq 4000	This study
*T. laevis*	ATCC 42080	–	USA	*Triticum* sp.	PRJNA393337	Illumina MiSeq	Nguyen et al. (2019)
*T. laevis*	DAOMC 238040	1997	Australia	*Triricum* sp.	PRJNA393335	Illumina MiSeq	Nguyen et al. (2019)
*T. laevis*	LLFL (CBS 144826)	2015	Germany	*Triticum aestivum*	PRJEB40624	Illumina HiSeq 4000	This study
*T. laevis*	L-19 (CBS 145173)	–	–	*Triticum aestivum*	PRJEB40624	Illumina HiSeq 4000	This study

^a^Westerdijk Fungal Biodiversity Institute (CBS-KNAW; Utrecht, The Netherlands)

### High molecular weight nucleic acid extraction and whole-genome sequencing

Total genomic DNA from freeze-dried mycelium was extracted using a modified CTAB-based method (Brandfass and Karlovsky 2008) as follows: 30–50 mg lyophilized mycelium was pulverized in 2 mL reaction tubes with four 4 mm sterile tungsten carbide beads at 22 Hz for 50 s using a tissue lyser (Qiagen Tissuelyser II, Qiagen, Hilden, Germany). The bead-beating step was repeated twice. The reaction tubes were shaken vigorously between the two disruption steps to loosen the mycelium from the bottom of the tubes after bead beating.

The CTAB buffer (600 µL) was supplemented directly before use with 400 µg RNase (Carl Roth, Karlsruhe, Germany), 5 µL β-mercaptoethanol and 0.2 mg proteinase K (Carl Roth, Karlsruhe, Germany) and then added to the ground mycelium. The samples were incubated for 60 min at 65 ºC and 400 rpm in a thermoshaker (Eppendorf, Hamburg, Germany). One volume of phenol/chloroform/isoamylalcohol (25:24:1 v/v/v) was added (Carl Roth, Karlsruhe, Germany) to the mixture followed by 10 min of incubation on ice before centrifugation for 10 min at 13,000×*g* at room temperature. The upper phase was transferred to a new reaction tube and inverted a few times to mix with chloroform (v/v). The mixture was incubated at room temperature for 10 min before being separated by centrifugation at 13,000×*g* at room temperature for 10 min. Finally, the DNA was precipitated for 20 min at room temperature using 0.6 volume of isopropanol (Merck, Darmstadt, Germany). The obtained DNA pellet was washed twice with 70% (v/v) ethanol and dried at room temperature, before it was finally dissolved in 500 µL commercially available elution buffer (10 mM Tris–Cl, pH 8.5) (Qiagen, Hilden, Germany) at room temperature.

To digest the remaining RNA, 100 µg RNase (Carl Roth, Karlsruhe, Germany) were added to the extracted gDNA, the mixture was inverted multiple times and incubated for in total 1 h at 42 °C at 100 rpm. After 30 min 7 µg proteinase K (Carl Roth, Karlsruhe, Germany) was also added. RNA and proteins were removed by adding one volume of phenol/chloroform/isoamylalcohol (25:24:1 v/v/v), and DNA was precipitated by isopropanol (1:1 v/v) (Merck, Darmstadt, Germany) as described above. Polar fractions were retrieved through 13,000×*g* centrifugation. The obtained DNA pellet was washed twice with 70% (v/v) ethanol and resuspended in the elution buffer. DNA from different extraction replicates was pooled. The quality and quantity of the isolated DNA was measured with a Qubit® 3.0 Fluorometer (Thermo Fisher Scientific, Darmstadt, Germany) and stored at −20 ºC until shipping.

For whole-genome sequencing, the DNA from single teliospore cultures of eleven isolates was shipped to GATC biotech (Konstanz, Germany) for fragmentation, library preparation, and sequencing on an Illumina HiSeq 4000 platform (125 bp, paired-end reads). Whole-genome shotgun sequencing of one isolate (*T. controversa* OR) was additionally performed using a Pacific Biosciences (PacBio) RS II instrument, and P6-C4 chemistry. A total of seven Single Molecule Real-Time (SMRT) cells were sequenced for this isolate.

### Genome assembly

Trimmomatic v0.36 (Bolger et al. 2014) was used to trim adapters and low quality reads from Illumina data from 11 *Tilletia* isolates (ILLUMINACLIP:TruSeq3-PE.fa:2:30:10 LEADING:3 TRAILING:3 SLIDINGWINDOW:4:15 MINLEN:70 AVGQUAL:25). High-quality reads with minimum lengths of 70 bp for both reads and > 25 average quality were retained for further processing. PacBio reads of *T. controversa* (OR) were corrected using the filtered Illumina reads from the same species using Proovread v2.12 (Hackl et al. 2014). The Prooveread-corrected untrimmed PacBio reads were further corrected and trimmed using the self-correction and trimming method implemented in Canu v1.6 assembler (Koren et al. 2017). The Canu assembler was also used to construct a draft assembly of corrected-trimmed PacBio reads, which was subsequently scaffolded with Illumina paired reads using SSpace-Standard V3.0 (Boetzer et al. 2011) and PacBio reads that were corrected and trimmed by Illumina reads using SSpace-LongRead (Boetzer and Pirovano 2014)). The assembly statistics were generated using assemblathon_stats.pl (Author: Keith Bradnam, Genome Center, UC Davis) and CEGMA v2.5 (Parra et al. 2007) to assess genome completeness. In a separate approach, all the 10 *Tilletia* isolates were assembled, using the remaining Illumina reads employing Velvet assembler v1.2.10 (Zerbino and Birney 2008) (-scaffolding on). Several assemblies were generated for all the species at different *k*-mers. Assembly statistics and CEGMA completeness of all the assemblies were tabulated and for individual species the best assembly in terms of statistics and CEGMA completeness was manually chosen.

Additionally, the genome assembly and annotation files of one *T. caries*, two *T. controversa*, and two *T. laevis* isolates sequenced by Nguyen et al. (2019) were retrieved from the National Center for Biotechnology Information (NCBI) repository. In total, 16 genomes comprising five *T. caries*, seven *T. controversa*, and four *T. laevis* isolates were used in this study (Table 1).

### Species tree recognition

Genome assemblies used in the phylogentic analyses are listed in Additional file 1: Table 1. For the phylogenetic study, orthologous genes were selected according to a modified approach described by Pizarro et al. (2018). Every assembly was assessed for 303 single-copy genes of the hierarchical catalog of eukaryotic orthologs v9 (Waterhouse et al. 2013) using BUSCO (Benchmarking Universal Single-Copy Orthologs) version 3.0.2 (Simao et al. 2015) in the genomic mode. The putative gene regions identified by BUSCO were extracted. For duplicated genes, we used the sequence that had the higher similarity score to its BUSCO reference. Each BUSCO gene recovered from each of the 27 genomes was aligned using MAFFT V. 7 (Katoh et al. 2017) adopting the iterative refinement algorithms L-INS-i (-localpair -maxiterate 1000—adjustdirectionaccurately). In order to reduce the effect of missing data, alignments with more than 7% of missing data (lacking corresponding sequences in more than two isolates per locus) were removed resulting in 241 genes that could be used for the inferences. Ambiguous regions within each alignment were removed using Gblock v 0.91b (Castresana 2000) with the default parameters (strict).

Species tree inferences based on the multispecies coalescent model (Degnan and Rosenberg 2006) were done because individual phylogenetic analyses based on individual genes can result in different gene trees that differ from the true species tree (Rannala and Yang 2003). First, we constructed approximately-maximum-likelihood phylogenetic trees for every single gene individually using FastTree 2.1.11 (Price et al. 2010) implemented in Geneious version 8.1.2 (Biomatters Limited, Auckland, New Zealand). As nucleotide substitution the Generalized Time-Reversible (GTR) model was used with the optimized gamma20 likelihood distribution. The Accurate Species Tree ALgorithm II (ASTRAL-II) (Mirarab and Warnow 2015) was employed to summarize coalescent inferences resulting from all trees. Clade support was evaluated by computing the local posterior probability (LPP) (Sayyari and Mirarab 2016). The trees were visualized in FigTree v1.4.4 (tree.bio.ed.ac.uk/software/figtree/).

### Identification of repetitive regions, simple sequence repeats (SSRs), and transposable elements (TEs)

Draft genome sequences were used to identify SSRs, also known as microsatellites by using the tool MIcroSAtellite identification (MISA) (Beier et al. 2017). The search criteria were at least ten repeat units for mononucleotide, six repeats for dinucleotide, and five for tri-, tetra-, penta-, and hexanucleotide motifs. SSRs with less than 100 bp distance from each other were considered as compound. The relative abundance for each SSR type was calculated by the number of repeats per Mb of genome.

Transposable elements were identified computing TransposonPSI (Haas 2010) with default settings. The program employs PSI-BLAST search (Altschul et al. 1997) against a database of various collections of TE families to identify matching regions in the genome. Additionally, we used RepeatModeler version 1.0.11 (Smit et al. 2008–2019) with the default settings to create a library comprising de novo identified repetitive elements. RepeatModeler employs three de novo repeat finders of RECON (Bao and Eddy 2002), RepeatScout (Price et al. 2005), and Tandem Repeats Finder (Benson 1999). The number of identified TEs and SRRs (see above) was subtracted from the total number of repeats identified by RepeatModeler as unclassified repetitive elements.

The resulting library of RepeatModeler was used to mask respective elements in the target genome sequences using RepeatMasker 4.0.9 (Smit et al. 2013–2015). For different purposes (see below), we generated both soft-masking (i.e. repeats replaced by lowercase letters) and hard-masking (i.e. repeats replaced by N).

### Detection of single nucleotide polymorphisms (SNPs) and small insertions and deletions (indels)

For pairwise SNP and indel identification, we used hard-masked genomes generated by RepeatMasker. For each genome pair, SNPs and the total length of indels in the aligned regions were counted using dnadiff wrapper from MUMmer 3.0 package (show-snps-C) (Kurtz et al. 2004). Average nucleotide identities of one-to-one alignments were also obtained from dnadiff output.

### Gene model prediction

Genes in the newly sequenced genomes (n = 11) were predicted from the soft-masked assemblies while for the publicly available genomes we used the existing gene annotations. We used a combination of ab initio and homology-based approach for gene model prediction. Gene models were created first by the incorporation of multiple sources of evidence using Gene Model Mapper (GeMoMa pipeline: V1.6.2 beta) (Keilwagen et al. 2016, 2018). GeMoMa is a homology-based gene prediction program and uses RNA-Seq data to incorporate evidence for splice site prediction. Afterwards, BRAKER2 (Brůna et al. 2020), which utilizes the ab initio gene predictor Augustus 3.3.3 (Stanke et al. 2006) and GeneMark-ET 4.33 (Lomsadze et al. 2014) self-training algorithms were applied. To do so, publicly available genome sequences and structural annotations of *Acaromyces ingoldii*, *Ceraceosorus guamensis*, *Jaminaea rosea*, *Meira miltonrushii*, *Pseudomicrostroma glucosiphilum*, *Tilletiopsis washingtonensis* isolates (Kijpornyongpan et al. 2018) and *Ustilago maydis* (Kamper et al. 2006) were downloaded from GenBank as references (accession numbers are given in Additionalfile 1: Table 2). Additionally, three RNA-Seq datasets were derived from two different *T. caries* isolates (DAOMC 238032 and WSP 72095/517) and one *T. controversa* isolate (DAOMC 236426) were downloaded from GenBank (Additionalfile 1: Table 1).

For adapter clipping and read trimming of the RNA-Seq data, the utility program Trim Galore version 0.4.0 (Krueger 2012–2019) was employed (qval >  = PHRED 30, minimal read length of 50 bp). Trimmed reads were mapped to the assembled genome sequences using STAR version 2.4.0d-2 (Dobin et al. 2012) with default parameters. The two RNA-Seq datasets of *T. caries* (SRR2513861 and SRR3337311) were mapped to *T. laevis* assemblies because no RNA-Seq data was available for *T. laevis* and the two species are closely related.

Protein-coding exons were extracted from the seven reference genomes by GeMoMa module Extractor (part of GeMoMaPipeline) using the default parameters. The GeMoMa Extract RNA-Seq evidence (ERE) was used to extract intron boundaries of each target genome by utilizing RNA-Seq data (coverage = true). We permitted alternative transcripts. The rest of the parameters were set as follows: maximum intron length = 2500, tBLASTn = false, ambiguity = ambiguous, score = ReAlign, rename = no. Filtered predictions (start = ’M’ and stop = ’*’ sorting = score/AA >  = 0.50) file for each genome generated by GeMoMa was used with align2hints command to produce hint file for BRAKER2. The corresponding softmasked genome, the STAR RNA-Seq mapped file, and the GeMoMa hint file were used to run BRAKER2 (UTR = on) for each genome. The generate gene model by BRAKER2 was used for further analysis. To predict the coding regions of transfer RNA (tRNAs), tRNAscan-SE 2.0 (Lowe and Chan 2016) was used with eukaryotic sequence source in the default search mode.

To estimate the completeness of the gene model predictions, BUSCO was used. We used the lineage dataset for Fungi-OrthoDB9 (Zdobnov et al. 2016) in the proteome mode.

### Functional annotation of the predicted genes

Genome-wide annotation was done to relate putative biological functions to the predicted genes. To make functional annotation comparable between all draft genomes, we analyzed all 16 genomes used in this study. The putative functions were assigned to the predicted proteins through one-to-one orthology assignments by eggNOG-Mapper 5.0.0 (Huerta-Cepas et al. 2018) (one-to-one ortholog, auto taxonomic adjust mode). Only functional annotations derived from Eukaryote or fungal sequence sources were accepted. Functional descriptions of Gene Ontology (Go) terms (Ashburner et al. 2000; Gene Ontology 2015), Kyoto Encyclopedia of Genes and Genomes (KEGG) pathways and modules (Kanehisa et al. 2013), and COG/KOG functional categories (Levasseur et al. 2013; Galperin et al. 2014), and SMART/PFAM domains (Letunic and Bork 2018) were obtained using eggNOG-Mapper.

### Prediction of encoded carbohydrate-active enzymes (CAZymes), secreted proteins, and secondary metabolites

Carbohydrate-active enzymes derived from the draft genomes were predicted using the HMM-based-dbCAN server (HMMdb v8.0) with a cut-off E-value < 1e^−17^ (suggested for fungi) and coverage > 0.50 (Zhang et al. 2018). Out of this prediction, the potential plant cell wall degrading enzymes were classified for their substrate according to Kijpornyongpan et al. (2018) and Benevenuto et al. (2018).

Putatively secreted proteins (referred to secretome in their totality) were identified by the presence of a signal peptide and absence of transmembrane domains in the predicted proteomes of each genome according to the suggestions of (Min 2010). Briefly, the proteome of each draft genome was first screened by SignalP 5.0 (Almagro Armenteros et al. 2019). To check whether the prediction belonged to an integral membrane protein, transmembrane α-helix predictor TMHMM v. 2.0 (Krogh et al. 2001) in tandem was employed. Those signal-peptide-like proteins showing any transmembrane helix topology were filtered out. Additionally, the signal peptides were predicted using Phobius (Kall et al. 2007) webserver accessed on Sep. 2019 with default parameters. In the end, only those putative proteins containing signal peptides that had been predicted by both independent approaches were annotated as secretome. To predict the effector repertoire from the predicted secretome of each genome, EffectorP 2.0 (Sperschneider et al. 2018) accessed on Sep. 2019 was used.

The draft genome sequences were searched for secondary metabolites and biosynthetic gene clusters using the fungal version of antiSMASH 5.0 (antibiotics and Secondary Metabolite Analysis Shell) (Medema et al. 2011). Since genes of a cluster may be dispersed on different contigs, the presence, completeness and order of each gene cluster was validated by aligning Illumina reads of each isolate to a reference sequence from each gene cluster group according to a mapping approach described by Weber et al. (2019). Briefly, the reference sequence was selected for each gene cluster group based on either length or high sequence conservation among the different isolates. Illumina reads of each isolate (Additionalfile 1: Table 1) were trimmed using Trimmomatic version 0.36 (Bolger et al. 2014) with a 4:15 sliding window. The trimmed reads were aligned to the different gene cluster references using Bowtie v2.4.1 (Langmead and Salzberg 2012). SAMtools v1.10 (Li et al. 2009) was used for file conversion to bam, validation of read pairing information (fixmate), removal of reading duplicates (rmdup), removal of mapped singleton readings, and calculation of position-specific reading depth (depth). Read depths were plotted with R version 3.5.1 (R Development Core Team 2013).

### Orthologous gene identification and clustering

The OrthoMCL pipeline v 2.0 (Li et al. 2003) was used to identify clusters of orthologous genes among all 16 isolates of the three species (inflate = 1.8 and E-value = 1e^−10^). Input translated protein sequences of all predicted genes contained also alternative transcripts per gene. The OrthoMCL program applies all-against-all BLASTp to estimate similarities between proteins and identifies groups using Markov clustering algorithm. The output of the program was parsed by using a custom-made phyton script to define; (i) orthologous genes that were present in all isolates referred to shared genes at the interspecies level and core genome at intraspecies level; (ii) orthologues genes shared between all isolates of a species and absent in the others (species-specific genes); (iii) the accessory (at intraspecies level) or variable (at interspecies level) genes which were dispensable and not present in all genomes; (iv) singletons presented only in a single isolate (isolate-specific genes). Putative functional prediction of an orthology cluster was reported only when at least 75% of the genes within shared an identical annotation.

## Results

### Genome assembly and annotation of *T. caries*,* T. controversa*, and* T. laevis*

**Fig. 1:**
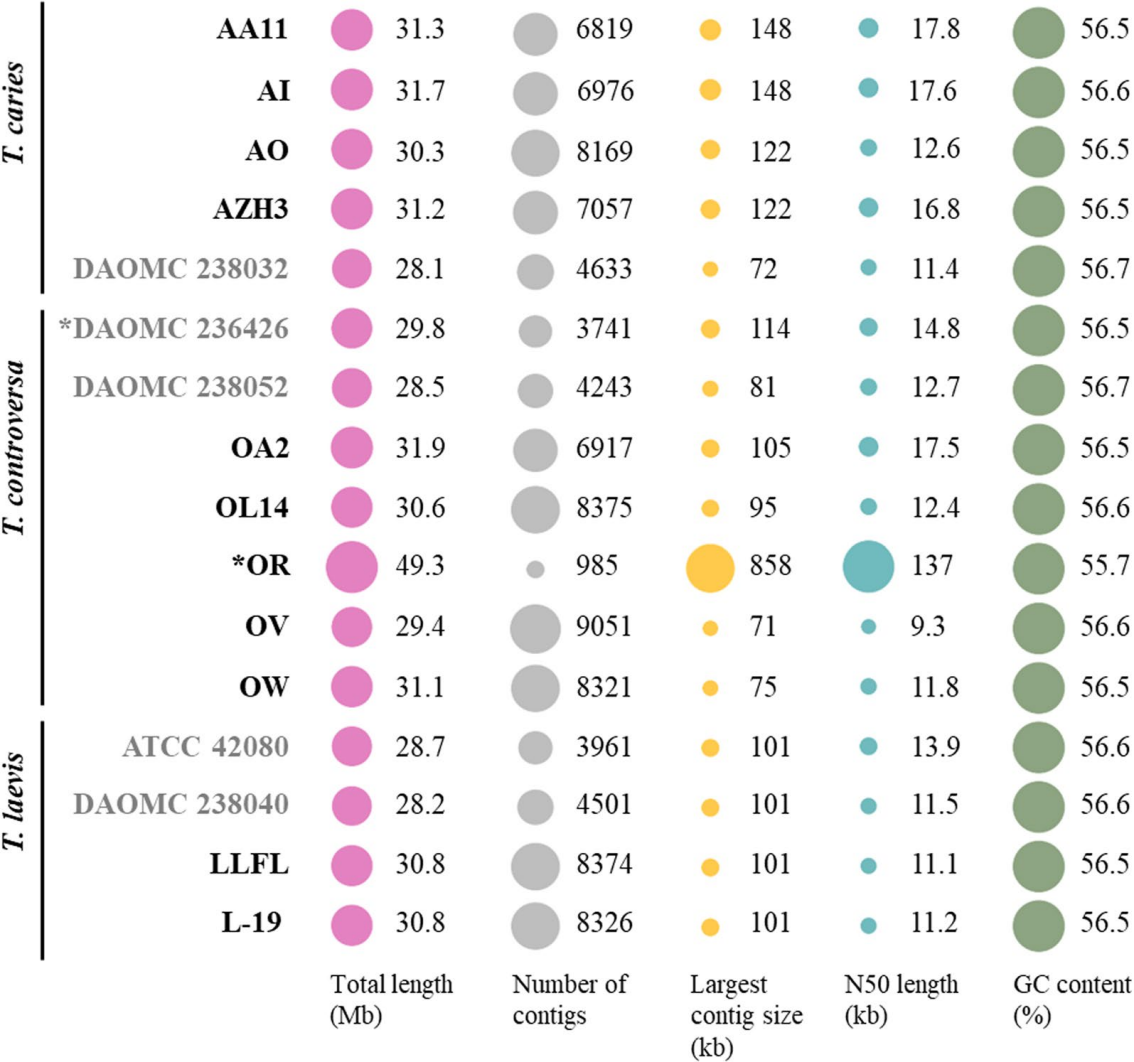
Bubble plots of descriptive numbers for each genome. The bubble sizes are scaled only within categories. The genome assemblies done in this work are presented in black font. Those in grey font relate to genomes published by Nguyen et al. (2019). Two *T. controversa* isolates OR and DAOMC 236426 were sequenced both on Illumina and PacBio platforms and marked with asterisks

The assembly of ten draft genomes based only on Illumina reads (four *T. caries*, four *T. controversa*, and two *T. laevis* isolates) resulted in assembled genome sizes of 30.3 to 31.7 Mb (*T. caries*), 29.4 to 31.9 Mb (*T. controversa*), and 30.8 Mb (*T. laevis*) with GC contents between 56.5 to 56.7% (Fig. 1). N50 values varied between 9.3 and 17.8 kb. The hybrid assembly of the *T. controversa* isolate OR, which was sequenced using both PacBio and Illumina reads, resulted in a draft genome size of 49.3 Mb (scaffold N50 = 137 kb) distributed in 985 scaffolds with a GC content of 55.7% (Fig. 1).

A combination of ab initio and order-specific gene model data was used after assessing annotation completeness for each annotation approach separately employing a genome (see the results in Additional file 1: Table 3). In total, 9,807 to 9,943 protein-coding genes were annotated in the *T. caries* genomes, 9,679 to 10,459 in the *T. controversa* genomes, and 10,160 and 10,203 in the two *T. laevis* genomes (Table 2). Coding sequences (CDS) consisted of 3.5 exons on average. Alternative splicing forms were predicted for up to 1.4% of the total CDS (Table 2). Genomes contained 110 to 178 genes encoding for tRNAs. The specificity of these tRNAs covered up to 48 of 61 possible anticodons and the codon usage was identical in all three species. To check whether the tRNA genes were clustered, we examined the location of tRNA genes in the most contiguous genome (OR isolate). A total of 178 putative tRNA genes were distributed over 102 scaffolds. The maximum number of 14 tRNA genes plus 9 pseudogenes spanned a 151,829 bp long region on scaffold number OR-9 (accession number CAJHJB010000889). The genome annotation completeness was estimated between 91.1 to 96.9% in the draft genomes (Table 2).

**Table 2:** Summary of gene model prediction results

Annotation statistics	*T. caries*				*T. controversa*					*T. laevis*	
	AA11	AI	AO	AZH3	OA2	OL14	OR*	OV	OW	LLFL	L-19
Gene model	9913	9943	9807	9930	9679	9956	10,459	9680	9797	10,203	10,160
tRNA	119	128	114	123	119	116	178	110	118	116	116
Average gene length (bp)	1790	1734	1756	1766	1813	1706	1931	1720	1755	1671	1674
Total CDS length (Mb)	14.9	14.8	14.5	14.8	14.8	14.6	16.9	14.1	14.5	14.6	14.6
Number of exons	36,009	35,788	35,117	35,601	35,266	34,951	40,922	33,870	34,789	35,521	35,439
Exons per gene	3.63	3.6	3.58	3.58	3.64	3.51	3.91	3.49	3.55	3.48	3.48
Number of introns	26,443	26,440	25,784	26,103	25,944	25,832	30,399	24,833	25,549	26,258	26,216
Introns per gene	2.66	2.65	2.62	2.62	2.68	2.59	2.9	2.56	2.6	2.57	2.58
% of genome covered by genes	56.3	54.1	56.5	55.8	54.7	55	40.5	56.3	55	55.03	54.86
Transcripts	10,052	10,069	9930	10,053	9800	10,066	10,585	9801	9914	10,334	10,292
Annotation completeness	96.6	95.8	93.8	95.1	95.5	94.5	96.9	91.1	93.8	93.1	94.1

*Reference genome

**Fig. 2:**
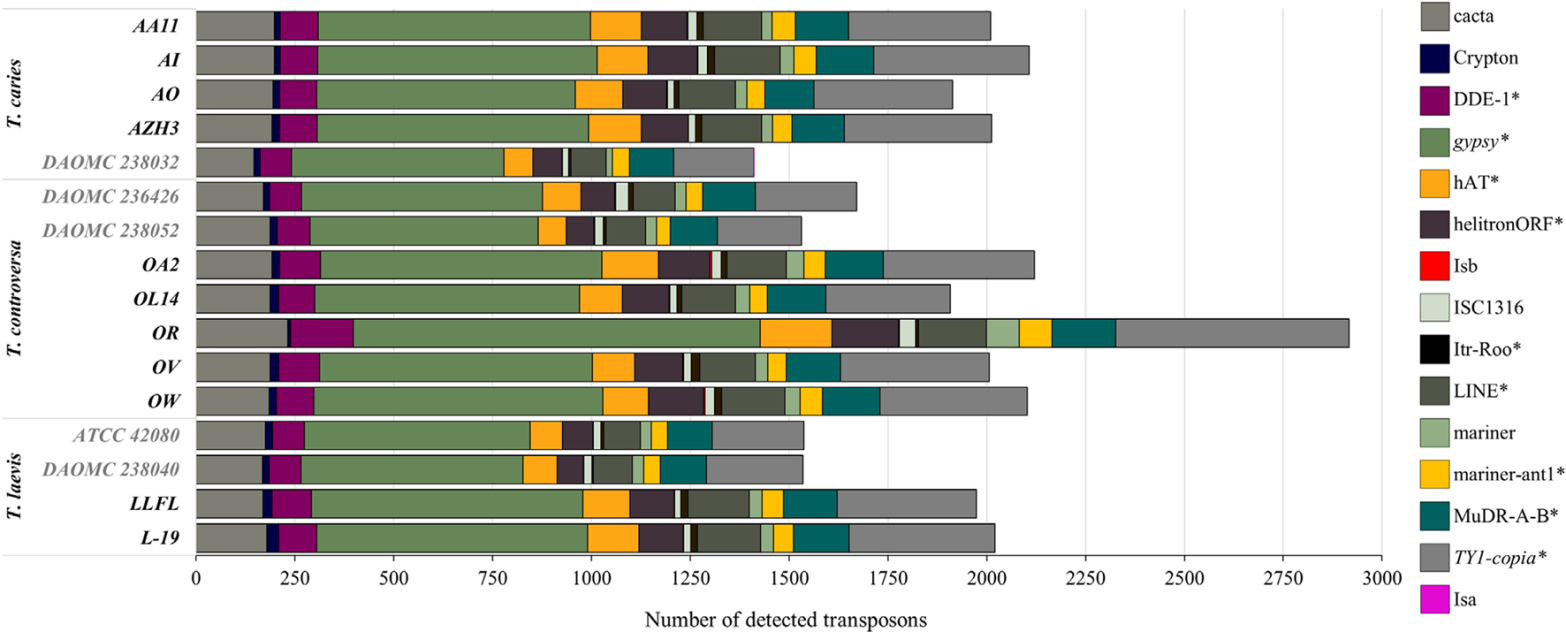
Number of detected transposable elements and their classification in 16 isolates of common and dwarf bunt. Significant differences in the total number of detected transposable elements between sequenced genomes of this study (black font) and publicly available ones are marked with * (Tukey HSD, α = 0.001)

Although repetitive elements had been generally regarded as junk DNA or remnants of molecular evolution, some repetitive sequences were shown to play diverse roles in environmental adaptation and genome evolution (Wöstemeyer and Kreibich 2002). The genome fraction assigned to repetitive elements in *Tilletia* species ranged from 7.8 to 13.7% for *T. caries*, 8.9 to 13.6% for *T. controversa*, and 9.1 to 11.8% for *T. laevis* (Additionalfile 1: Table 4) and overall, a higher proportion of repetitive elements was found in the newly sequenced genomes. Exceptionally, roughly a four times higher number of repetitive elements (37%) was revealed in the genome sequence of *T. controversa* isolate OR and its 49.3 Mb assembled genome size. Transposition is one of the causes of genomic plasticity and plays an important role in pathogenicity and adaptive evolution (Casacuberta and González 2013; Muszewska et al. 2019; Razali et al. 2019). Transposable elements made up to 3.7% of all repetitive elements in the studied genomes. The values were very similar for all three species. Transposable elements can move or copy from one locus to another and are classified based on their mode of dispersion (Levin and Moran 2011). The detected TEs were classified into one of 15 superfamilies (Fig. 2), of which DDE-1, *gypsy*, hAT, helitronORF, Itr-Roo, Line, mariner-ant1, MuDR-A-B, and TY1-*copia* were more prevalent in the genomes of European isolates compared to the North American genomes. In all isolates, regardless of the sequencing platform used, the *gypsy*-like and TY1-*copia*-like superfamilies were most common, accounting for more than half of the total TEs in each genome (Fig. 2). Additionally, we classified a total of 6,564 to 10,031 repetitive elements as SSRs (Additionalfile 1: Table 5), accounting for 0.53 to 0.63% of the entire genomes. Trinucleotide SSRs (35.2 to 42.8% of all SSRs) were the most abundant.

### Phylogenetic analysis

**Fig. 3:**
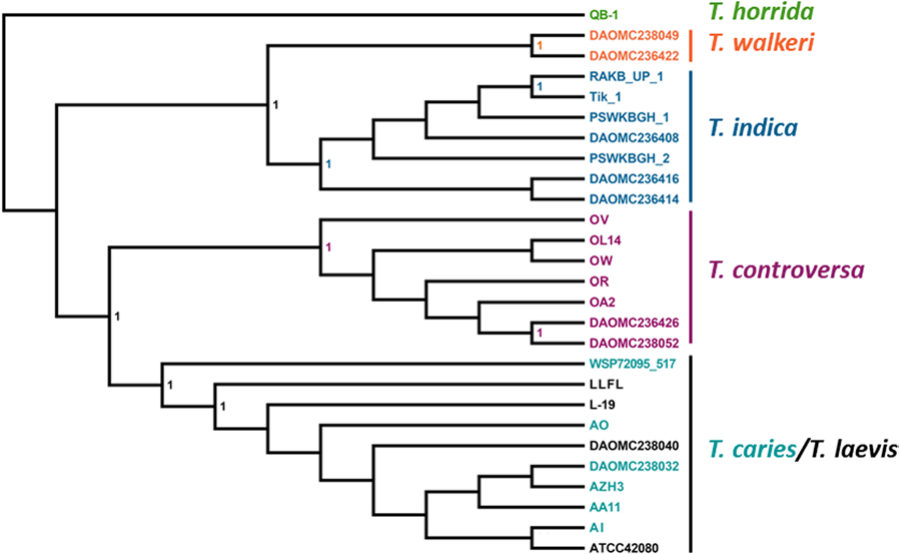
Coalescence-based phylogenetic analysis on a set of 241 genes and 27 strains of six *Tilletia* species. Individual trees were obtained from approximately maximum likelihood analysis per locus and the coalescence inference was based on all the resulting trees using ASTRAL II. Each isolate is color coded based on species identification. Node support values are given as local posterior probabilities. Only supporting values above 0.95 are indicated at nodes

From a total of 303 initial BUSCO genes, 241 were used for the construction of individual gene trees on which the multispecies coalescence inferences of 27 strains representing six *Tilletia* species were based on. In the phylogenetic tree (Fig. 3) the sequenced strains clustered in highly supported groups according to their species circumscription with one exception: The strains of *T. caries* and *T. laevis* clustered intermingled in a polyphyletic manner within a joint monophyletic group suggesting their conspecificity.

### Genomic synteny and genome-wide diversity

**Fig. 4:**
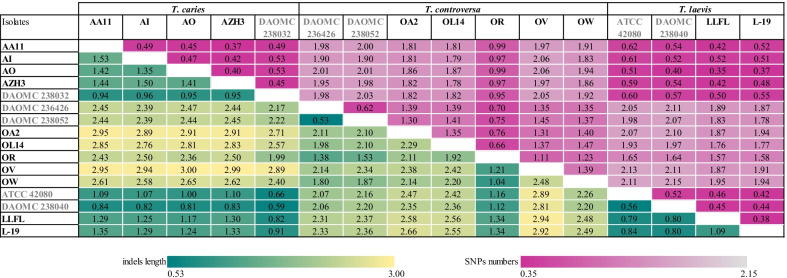
The number of SNPs per kb (upper triangle) and the total length of indels per kb (lower triangle) in the repeat masked genomes of 16 isolates of common and dwarf bunt using NUCmer. The isolates and species are given as row and column labels where the sequenced genomes in this study are presented in black. Density of the SNPs and indels are represented by two color scales

Overall, 82.7 to 94.3% of the assembled genomes could be aligned pairwisely with an average nucleotide identity between 98.7 to 99.6% in one-to-one aligned regions, excluding repetitive sequences (Additionalfile 1: Table 6). Based on the number of SNPs and the total length of indels within species, *T. laevis* isolates, with max. 0.52 SNPs/kb and 1.09 bp indels/kb, were the most homogeneous, while *T. controversa* showed the greatest degree of nucleotide diversity (max. 1.47 SNPs/kb and 2.48 indels bp/kb) (Fig. 4). On the interspecies level, low nucleotide variation was observed between *T. caries* and *T. laevis* species while all isolates of these two species exhibited a greater distance to the isolates of *T. controversa* (Fig. 4). No correlation between the sequencing platform and genomic diversity was observed.

### Functional genomics

**Fig. 5:**
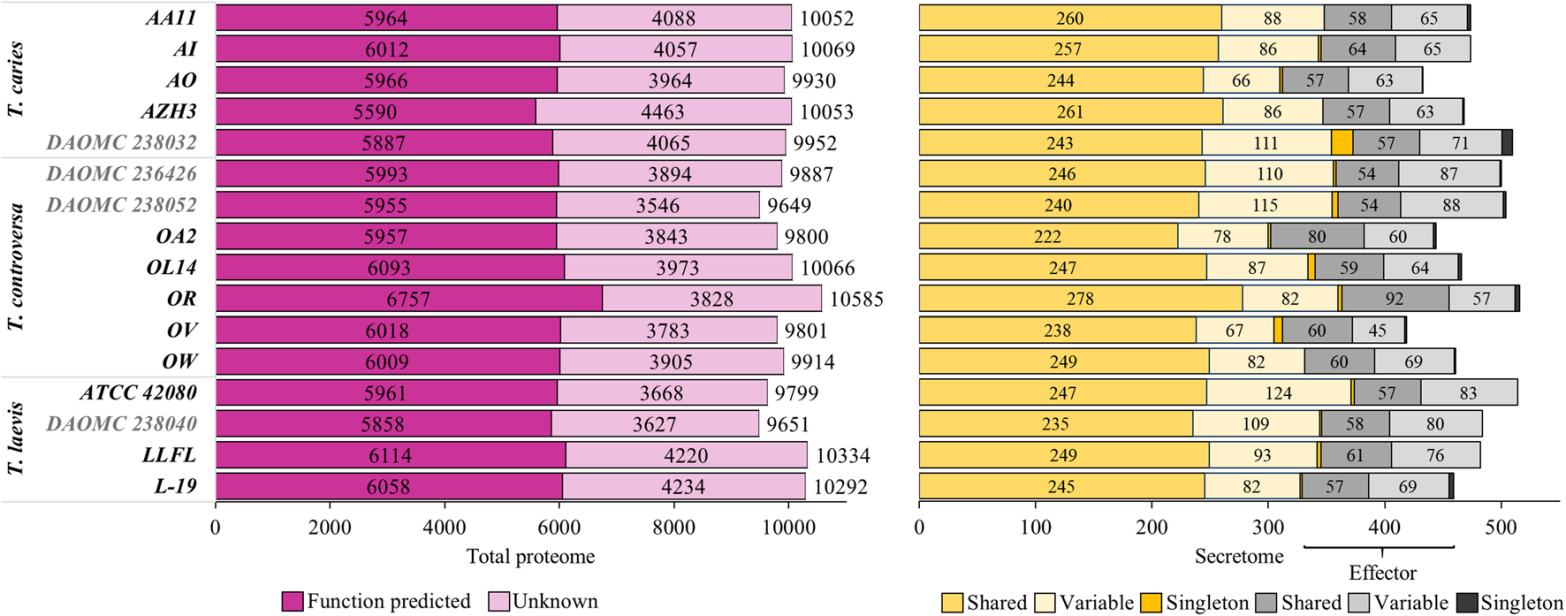
In silico functional genomic prediction. A: Stacked histograms of functional annotation results of each genome. B: Total predicted secretome and effectors and their distribution in different orthology clusters at the interspecies level. Shared fraction refers to the secreted proteins and effectors which are shared between all 16 isolates. Variables are represented in an absent-present manner in different genomes. Secretome and effectors detected in singletons are isolate specific. Values shown are the total numbers of proteins

Functional information was assigned to gene products based on protein sequence homology. To ensure comparability, functional annotation was performed for the protein-coding genes of all genomes including new functional annotation of published genomes. At least 55.5% of all coding sequences in each genome were functionally annotated (Fig. 5A). In general, the identified biological pathways and functional categories were remarkably similar across the three species.

To overcome plant defense systems for successful colonization, plant pathogens employ plant cell wall-degrading enzymes (PCWDE) that are part of the carbohydrate-active enzymes, effectors which are a subgroup of secreted proteins, and secondary metabolites (Kimura et al. 2001; Chisholm et al. 2006). We searched the predicted proteomes for these important modulators as described below:

#### Carbohydrate-active enzymes

Carbohydrate-active enzymes are responsible for the biosynthesis, modification, and breakdown of complex carbohydrates and glycoconjugates (Cantarel et al. 2009). Different CAZyme classes comprising of Glycoside Hydrolases (GHs), Polysaccharide Lyases (PLs), Carbohydrate Esterases (CEs), Glycosyltransferases (GTs), Auxiliary Activities (AAs), and Carbohydrate-Binding Modules (CBMs) were searched in each of the predicted proteomes. Similar numbers of CAZymes were predicted in the genomes of *T. caries* (up to 212), *T. controversa* (up to 213), and *T. laevis* (up to 209). The predicted CAZymes belonged to 72 families and 22 subfamilies (Additionalfile 1: Table 7), of which GH followed by GT were most prominent (74 to 96 and 45 to 48 genes, respectively). All three species possessed a limited number of CAZyme genes for starch/glycogen metabolism. Starch can presumably be metabolized by the activities of GH13, GH37, GH65 (Trehalase), and also by GH15, GT3, and CBM21, which are involved in glycogen hydrolases.

Thirteen to 20 enzymes assigned to six families and seven subfamilies were referred to as PCWDEs (Additionalfile 1: Table 7). Although the majority of main CAZymes involved in cellulose breakdowns such as GH6, GH7, GH12, GH44, AA9, and CMB1 were absent in common and dwarf bunt, an expansion of the GH45 family was observed. The GH45 family is composed mainly of β-1,4-endoglucanases which play a role in the hydrolysis of soluble β-1,4 glucans of plant cell wall polysaccharides such as cellulose. More than half of the predicted PCWDEs were assigned to this family. Harboring genes putatively encoding peroxidases of the family AA2 indicates that the three *Tilletia* species may have a capacity to degrade lignin.

#### Secreted proteins, effectors, and genes related to pathogenicity, life cycle, and infection

Secreted proteins modulate interactions of plant pathogenic fungi with their hosts and decisively affect the outcome of the interaction (Girard et al. 2013; Delaunois et al. 2014; Kim et al. 2016). The size of the predicted secretomes in *T. caries* (433 to 510), *T. controversa* (419 to 516), and *T. laevis* (459 to 514) (Fig. 5B) was comparable. Effectors are a subset of the secretome that can suppress plant defense against infection by manipulating the plant’s immune system (Kamoun 2006, 2007; Dodds et al. 2009; Hogenhout et al. 2009). Among the secreted proteins, 107 to 144 effector-like proteins were predicted. Effectors can be localized in a cluster (Brefort et al. 2014). A total of 123 putative effectors were distributed across 75 scaffolds in the genome of the *T. controversa* isolate OR, which was sequenced using both PacBio and Illumina platforms. Maximum five putative effectors were detected on a scaffold spanning a 23,909 bp region, indicating that the genes were not located in clusters.

Functionally characterized proteins involved in pathogenicity, life cycle, and infection in *U. maydis* and a few other smut fungi had mostly no orthologs in the proteome of common and dwarf bunt (Table 3). Out of 56 sequences of functionally characterized proteins, only six putative orthologous genes were identified by a tBLASTn search (at least 60% coverage and > 20% identity). These genes included (1) Clp1 required for the proliferation of dikaryotic filaments *in planta* (Scherer et al. 2006); (2) defense-suppressing virulence effector Pep1 (Doehlemann et al. 2009; Hemetsberger et al. 2012, 2015; Sharma et al. 2018); (3) Pep4 involved in dimorphism and pathogenesis (Soberanes-Gutiérrez et al. 2015); (4) precursor of the peptide conferring surface hydrophobicity to hyphae Rep1 (Wösten et al. 1996; Müller et al. 2008; Teertstra et al. 2009); (5) peroxisomal sterol carrier protein Scp2 (Krombach et al. 2018); and (6) high-affinity sucrose transporter required for virulence Srt1 (Wahl et al. 2010).

**Table 3:** Functionally characterized genes involved in pathogenicity, life cycle, and infection

Gene ID	Gene name	Source species	Putative orthologs	References
smut_2965		*Tilletia horrida*	NA	Wang et al. (2020)
smut_5844		*T. horrida*	NA	Wang et al. (2020)
sr10077	*SAD1*	*Sporisorium reilianum*	NA	Ghareeb et al. (2015)
UHOR_10022	*UhAvr1*	*Ustilago hordei*	NA	Ali et al. (2014)
UMAG_00715	*stp3*	*U. maydis*	NA	Lanver et al. (2018)
UMAG_01235		*U. maydis*	NA	Skibbe et al. (2010)
UMAG_01236		*U. maydis*	NA	Skibbe et al. (2010)
UMAG_01238		*U. maydis*	NA	Skibbe et al. (2010)
UMAG_01240		*U. maydis*	NA	Skibbe et al. (2010)
UMAG_01241		*U. maydis*	NA	Skibbe et al. (2010)
UMAG_01375	*Pit2*	*U. maydis*	NA	Doehlemann et al. (2011)
UMAG_01829	*afu1*	*U. maydis*	NA	Lanver et al. (2018)
UMAG_01987	*Pep1*	*U. maydis*	YES	Doehlemann et al. (2009)
UMAG_02012	*ApB73*	*U. maydis*	NA	Stirnberg and Djamei (2016)
UMAG_02135	*eff1-5*	*U. maydis*	NA	Khrunyk et al. (2010)
UMAG_02136	*eff1-6*	*U. maydis*	NA	Khrunyk et al. (2010)
UMAG_02137	*eff1-7*	*U. maydis*	NA	Khrunyk et al. (2010)
UMAG_02138	*eff1-8*	*U. maydis*	NA	Khrunyk et al. (2010)
UMAG_02140	*eff1-10*	*U. maydis*	NA	Khrunyk et al. (2010)
UMAG_02141	*eff1-11*	*U. maydis*	NA	Khrunyk et al. (2010)
UMAG_02239	*See1*	*U. maydis*	NA	Redkar et al. (2015a), Redkar et al. (2015b)
UMAG_02374	*Srt1*	*U. maydis*	YES	Wahl et al. (2010)
UMAG_02438	*Clp1*	*U. maydis*	YES	Scherer et al. (2006)
UMAG_02473		*U. maydis*	NA	Skibbe et al. (2010)
UMAG_02474		*U. maydis*	NA	Skibbe et al. (2010)
UMAG_02475	*Stp1*	*U. maydis*	NA	Schipper (2009)
UMAG_03172	*Rbf1*	*U. maydis*	NA	Heimel et al. (2010)
UMAG_03223	*Mig1*	*U. maydis*	NA	Basse et al. (2000)
UMAG_03274	*rsp3*	*U. maydis*	NA	Ma et al. (2018)
UMAG_03313	*eff1-3*	*U. maydis*	NA	Khrunyk et al. (2010)
UMAG_03314	*eff1-4*	*U. maydis*	NA	Khrunyk et al. (2010)
UMAG_03924	*rep1*	*U. maydis*	YES	Teertstra et al. (2009)
UMAG_04926	*pep4*	*U. maydis*	YES	Soberanes-Gutiérrez et al. (2015)
UMAG_05295		*U. maydis*	NA	Skibbe et al. (2010)
UMAG_05297		*U. maydis*	NA	Skibbe et al. (2010)
UMAG_05299		*U. maydis*	NA	Skibbe et al. (2010)
UMAG_05300		*U. maydis*	NA	Skibbe et al. (2010)
UMAG_05301		*U. maydis*	NA	Skibbe et al. (2010)
UMAG_05302	*Tin2*	*U. maydis*	NA	Skibbe et al. (2010), Brefort et al. (2014)
UMAG_05304		*U. maydis*	NA	Skibbe et al. (2010)
UMAG_05305		*U. maydis*	NA	Skibbe et al. (2010)
UMAG_05306		*U. maydis*	NA	Skibbe et al. (2010)
UMAG_05307		*U. maydis*	NA	Skibbe et al. (2010)
UMAG_05308		*U. maydis*	NA	Skibbe et al. (2010)
UMAG_05311		*U. maydis*	NA	Skibbe et al. (2010)
UMAG_05312		*U. maydis*	NA	Skibbe et al. (2010)
UMAG_05313		*U. maydis*	NA	Skibbe et al. (2010)
UMAG_05314		*U. maydis*	NA	Skibbe et al. (2010)
UMAG_05318	*Tin4*	*U. maydis*	NA	Skibbe et al. (2010), Brefort et al. (2014)
UMAG_05319	*Tin5*	*U. maydis*	NA	Brefort et al. (2014)
UMAG_05731	*Cmu1*	*U. maydis*	NA	Djamei et al. (2011)
UMAG_06098	*UmFly1*	*U. maydis*	NA	Ökmen et al. (2018)
UMAG_10067	*stp2*	*U. maydis*	NA	Lanver et al. (2018)
UMAG_10556	*Tin3*	*U. maydis*	NA	Brefort et al. (2014)
UMAG_11938	*Scp2*	*U. maydis*	YES	Krombach et al. (2018)
UMAG_12197	*cce1*	*U. maydis*	NA	Seitner et al. (2018)

#### Biosynthesis of secondary metabolites

**Fig. 6:**
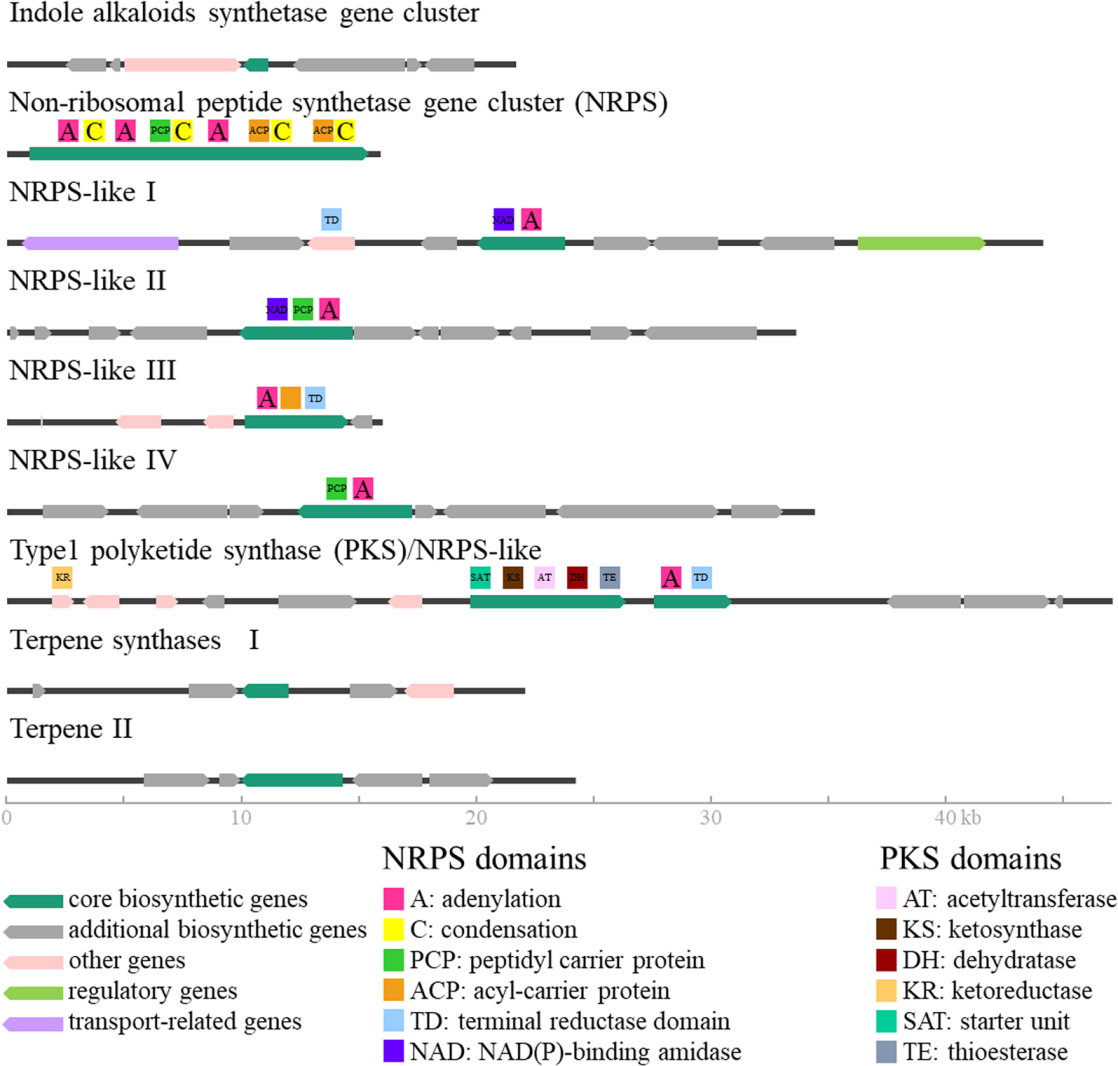
The gene organization of the nine putative secondary metabolite gene clusters found in all 16 T*. caries*, *T. controversa*, and *T. laevis* isolates

Secondary metabolites (syn. specialized metabolites) are compounds of low-molecular weight, which are typically species-specific and are believed to possess ecological functions (Pusztahelyi et al. 2015; Macheleidt et al. 2016). Nine putative secondary metabolite biosynthesis clusters comprising 62 genes were identified, which were highly conserved (> 95% identity) across all three species (Fig. 6 and Additionalfile 1: Table 8). Eight out of these nine secondary metabolite gene clusters were recovered almost completely in the different draft genomes by aligning Illumina reads to the respective cluster sequences (Additionalfile 2: Fig. 1). Of those clusters two different clusters encoded for terpene synthases, one cluster was putatively responsible for the synthesis of indole alkaloids, four clusters contained non-ribosomal peptide like synthetases (NRPS-like) genes, and one cluster comprised an NRPS gene. The last secondary metabolite gene cluster was a hybrid cluster encoding for a type 1 polyketide synthases (T1PKS)/NRPS-like and was only partially found in most of the tested isolates. Two highly similar NRPS-like gene clusters were exclusively identified in the *T. controversa* isolate OR (cluster V in Additionalfile 1: Table 8).

The translated sequences of each of the core NRPS and NRPS-like genes were searched for the adenylation domains according to NRPSpredictor2 (Röttig et al. 2011) which predicts the amino acid substrate based on the Stachelhaus codes (Stachelhaus et al. 1999). The results are presented in Additionalfile 1: Table 9. The only predicted NRPS gene cluster, which potentially encodes three modules, that are possibly used twice to synthesize a hexapeptide, may participate in siderophores production because the adenylation domains showed 45 to 53% identity to the *U. maydis* ferrichrome siderophore peptide synthetase.

#### *Genomic insight into trimethylamine synthesis in Tilletia* spp.

Fishy smell of grains infected with smut is caused by trimethylamine (TMA), which was isolated already in 1887 from ergot (*Claviceps purpurea*) and *Ustilago* sp. (Diehl 1887) and in 1932 from *T. laevis*-infected wheat (Hanna et al. 1932). It is not known how fungi synthesize TMA while the biosynthesis of TMA in bacteria was unraveled (Craciun and Balskus 2012). The precursor of TMA in bacteria is choline and the reaction is catalyzed by choline trimethylamine-lyase CutC, which is activated by activating protein CutD. The sequences of both proteins are highly conserved (Martínez-del Campo et al. 2015). Based on the report that TMA in ergot also originates from choline (Brieger 1887), we assumed that TMA biosynthesis in bacteria and fungi follow a convergent path or the pathway was transferred from bacteria to fungi, as was reported for other genes (Jaramillo et al. 2015; Navarro-Muñoz and Collemare 2020). We therefore searched for homologs of *cut*C in *Tilletia* spp. No such protein was found in the proteome of common and dwarf bunt, indicating that *Tilletia* spp. do not possess choline trimethylamine-lyase. Searches for proteins similar to activating protein CutD failed, too. Both genes were also missing from the genomes of *Ustilago* and *Claviceps*, indicating that the biosynthesis of TMA in smut and ascomyceteous fungi is different from bacteria.

Ethanolamine is a structural analog of choline. In bacteria, the degradation of ethanolamine to ammonia is catalyzed by vitamin B_12_-dependent ethanolamine ammonia-lyase EutBC (Garsin 2010). This enzyme inspired the search for choline degradation pathway that eventually led to the discovery of CutC/CutD (Craciun and Balskus 2012). We searched *Tilletia* genomes for genes similar to *eut*BC, too, but no such gene was found, indicating that the synthesis of TMA in fungi does proceed by the removal of the hydroxyethyl group from choline by an enzyme related to ethanolamine ammonia-lyase.

### Inter- and intraspecies variation of protein-coding genes

**Fig. 7:**
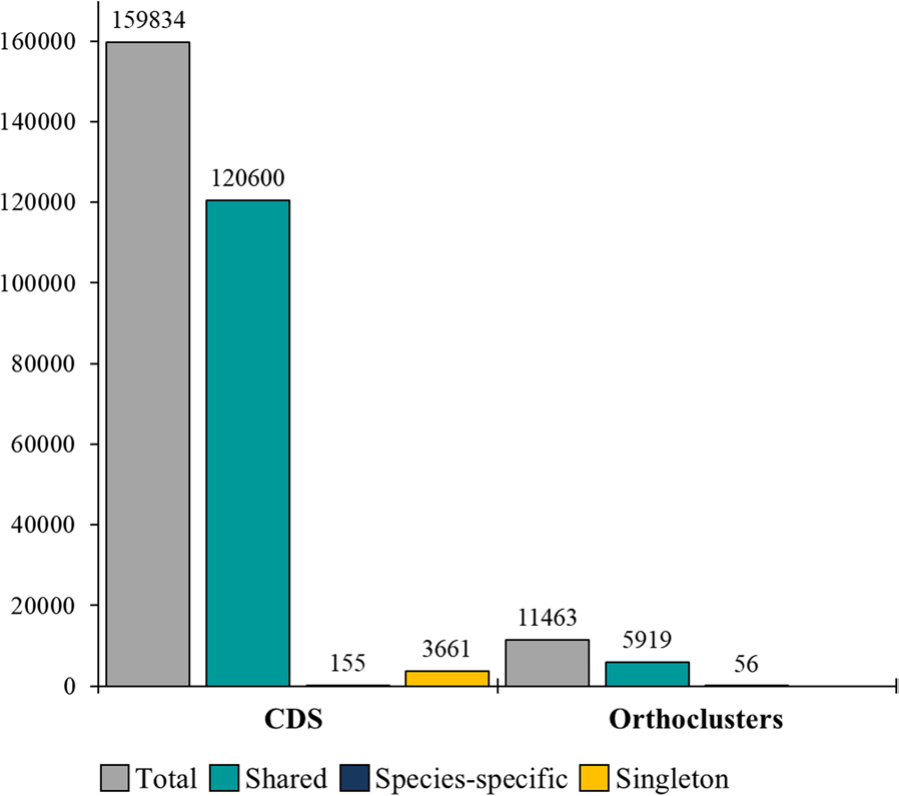
Distribution of orthologues clusters and CDS among 16 isolates of *T. caries*, *T. controversa*, and *T. laevis* species. Out of 159,834 total CDS, the majority (120,600) which clustered in 5,919 orthology clusters were shared between all the 16 isolates

**Fig. 8:**
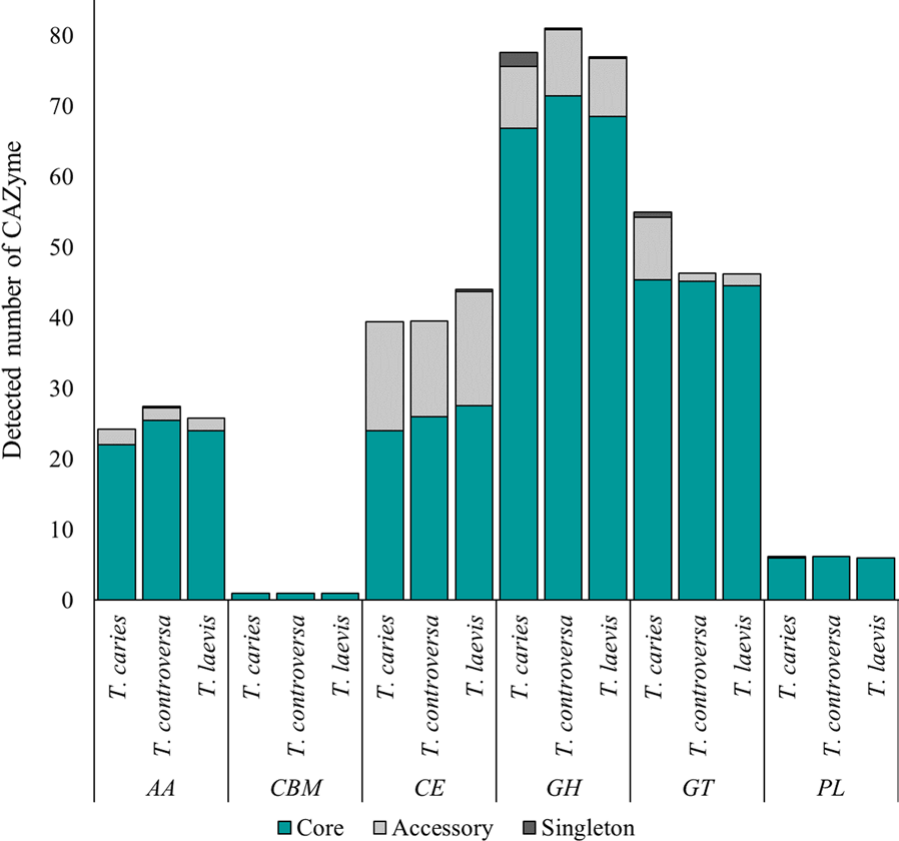
Distribution of putative CAZymes in the shared and variable orthology clusters, and singleton genes between five isolates of *T. caries*, seven isolates of *T. controversa*, and four isolates of *T. laevis*. The majority of the CAZymes are conserved and shared between the three species. Glycoside Hydrolases (GHs), Polysaccharide Lyases (PLs), Carbohydrate Esterases (CEs), Glycosyltransferases (GTs), Auxiliary Activities (AAs), and Carbohydrate-Binding Modules (CBMs)

To compare protein-coding genes within and among the three species, all 159,834 predicted CDS were grouped into orthology clusters based on the sequence homology of their products. From the total of the CDS, 97.6% were grouped into 11,463 orthology clusters; the remaining 2.4% genes were singletons that did not group to any orthology cluster (Fig. 7, Additionalfile 1: Table 10). A total of 5,919 orthology clusters (comprising 75.4% of total CDS) were shared by all 16 isolates. Many of them (4,167) were single-copy genes that did not have any paralog in any isolate. Additional 3,203 orthology clusters were shared among all species by at least one but not all isolates per species, indicating that these genes were neither essential nor species-specific (Additionalfile 1: Table 10). Interestingly, 84% of the total predicted CAZymes (Fig. 8), 72.5% of the total secretome (Fig. 5B), and 47.4% of the genes encoding effectors (Fig. 5B) were among the 5,919 orthology clusters shared and conserved across all species.

The number of species-specific orthology clusters defined by CDS that were present in all isolates of the target species only, but not in any isolate of the other species varied between 1 (*T. caries*), 21 (*T. controversa*), and 3 (*T. laevis*) (Additionalfile 1: Table 11). With a more relaxed definition, allowing the gene of an orthology cluster to be missing at most in one isolate of the target species, the numbers increased to 7, 39, and 10 in *T. caries*, *T. controversa*, and *T. laevis*, respectively. We were also interested in the genes that were present in both causal agents of common bunt (*T. caries* and *T. laevis*) only. Using the strict definition, we found 19 common bunt-specific orthology clusters. Under relaxed criteria, allowing an orthology cluster to be missing in at most two isolates, we found 40 common bunt-specific orthology clusters. Putative functions were assigned to only 16 out of 96 total common and dwarf bunt-specific orthology clusters (relaxed and strict) (Additionalfile 1: Table 11). In total, species-specific orthology clusters comprised only 0.09% of the total CDS (Fig. 7). Not a single effector nor CAZyme was detected among the species-specific genes. However, two orthology clusters comprised genes that putatively encode secreted proteins. One of them was specific for *T. controversa* and the other for the common bunt species *T. caries* and *T. laevis*. A more specific functional prediction of these interesting genes was not possible.

Based on the orthology clusters, we assigned CDS to core genomes (present in all isolates of a species) and pan-genomes (all CDS present in at least one isolate of a species) of each species individually. The largest core genome belonged to *T. laevis* (95.4% of the pan-genome), while *T. controversa* had the largest accessory genome (6.1% of the pan-genome) (Table 4).

**Table 4:** Pan-genomes of *T. caries*, *T. controversa*, and *T. laevis*

Species	Isolates	Pan-genome		Core genome			Accessory genome			Singleton	
		CDS	Orthology cluster	CDS	Orthology cluster	%	CDS	Orthology cluster	%	CDS	%
*T. caries*	5	50,056	9187	46,492	7020	92.8	2238	2167	4.5	1284	2.5
*T. controversa*	7	69,702	9732	63,728	6238	91.4	4297	3494	6.1	1677	2.4
*T. laevis*	4	40,076	9153	38,264	7200	95.4	1187	1953	2.9	625	1.5

## Discussion

### Insights into the genomic features of common and dwarf bunt

In this study, whole-genome sequencing, assembly, as well as structural and functional annotation of 11 isolates of *T. caries*, *T. controversa*, and *T. laevis* were performed. We additionally included the assembled genomes of five isolates of these species, which were previously sequenced by Nguyen et al. (2019) to assess the inter- and intraspecific genetic variation of these important wheat pathogens. Additionally, phylogenetic analyses were performed to obtain an evolutionary framework for interpretation of the results.

The size of the assembled genomes of ten *Tilletia* isolates based on Illumina reads ranged from 29.4 to 31.9 Mb. These numbers were similar to those obtained by the genome analyses done by Nguyen et al. (2019) who reported genome sizes of 28.1 to 29.9 Mb. A significantly broader range of genome sizes was reported by Russell and Mills (1993) applying an electrophoretic karyotype analysis, where genome sizes were estimated in the range of 28–39 Mb for *T. caries* and 34–40 Mb for *T. controversa*, distributed on 14–20 chromosomes in *T. caries* and 19–20 chromosomes in *T. controversa*, respectively. The genome of one isolate of *T. controversa* (OR) was assembled using a combination of PacBio and Illumina reads. This approach resulted in the fewest scaffolds (985 scaffolds) and an increase of N50 to 137 kb. In addition, as more repetitive DNA could be resolved using the long PacBio reads, the assembled genome size increased significantly (49.3 Mb) This higher sequencing depth also resulted in almost 50% more tRNA identification compared to the other isolates.

It is known that the proportion of repetitive regions can differ significantly between closely related species as is exemplified by *Fusarium oxysporum* and *F. graminearum* where repetitive elements account for 16.83 Mb and 0.24 Mb respectively (Ma et al. 2010). In our study, the percentage of repetitive regions was variable among the 15 isolates (7.8 to 13.7%) without resulting in a difference between the three species. Moreover, the true proportion of repetitive regions can be expected to be higher as exemplified by the number of 37% found in the isolate OR. This observation suggests that a variable number of repeat elements might have collapsed in the assemblies based on short reads and demonstrates the importance of long reads. The transposon types *Gypsy* followed by *Copia*, both belonging to the long terminal direct repeats (LTR) class of retrotransposons, were the most abundant in all three bunt species. Similarly, *Gypsys* were the most frequent TE reported in *T. indica* (Gurjar et al. 2019; Mishra et al. 2019) as well as in *T. horrida* (Wang et al. 2018). *Gypsy*s are the most successful group of TEs in fungi (Gorinsek et al. 2004) and plants (Sabot and Schulman 2006) and are capable to increase their number by autonomous transposition (Elliott and Gregory 2015).

#### Carbon utilization and establishment of fungal biotrophy

As other biotrophic pathogens, *T. caries*, *T. controversa*, and *T. laevis* encode for a relatively low number of CAZymes (Zhao et al. 2013; Lyu et al. 2015). Moreover, the three species were quite similar in the diversity and abundance of identified CAZyme families. Plant-parasitic fungi secrete a variety of CAZymes, which may play a role in pathogenicity and virulence and are needed to successfully degrade plant cell walls and to complete host invasion (Annis and Goodwin 1997; Gibson et al. 2011; Kubicek et al. 2014). Based on the in silico analyses, all 16 isolates had two putatively secreted CAZymes in common; one belonged to one chitin deacetylases of the family CE4 and one to the family GH152. CE4 is suggested to play a role in the modification of fungal cell walls for masking hyphae to escape from enzymatic hydrolysis by host chitinases through de‐N‐acetylation of chitin (El Gueddari et al. 2002; Boneca et al. 2007). The enzyme β-1,3-glucanase (GH152) is suggested to play a role in cell wall softening during morphogenesis (Mouyna et al. 2013). Common and dwarf bunt, similar to *T. indica* (Gurjar et al. 2019), have genes encoding for GH8 (broad activity hydrolase), which was suggested to be present in all Ustilaginomycotina (Kijpornyongpan et al. 2018). Interestingly, *Tilletia* spp. harbored families coding for PL14 and AA2 enzymes that are involved in lignin decomposition, which were completely absent in other studied Ustilaginomycotina, but present in Agaricomycotina (Kijpornyongpan et al. 2018) where the majority of lignin decomposing white-rot fungi belong to. Putative genes encoding for PL14 and AA2 are also reported from *T. indica* CAZyme analyses (Gurjar et al. 2019). The role of these genes in *Tilletia* is puzzling.

Almost half of the putative genes encoding for effectors in the multi-species comparison of *T. caries, T. controversa,* and *T. laevis* were among the variable genes (dispensable and not present in all genomes), which is in agreement with their ability to undergo rapid diversification including duplication, deletions, and point mutations (Oliver and Solomon 2010; Rouxel et al. 2011). Interestingly, most of the genes that have been functionally characterized in the model fungi were either absent or poorly conserved in the genomes of the common and dwarf bunt agents. Those which had orthologoues in the genome of the three species were suggested to be either essential for establishing biotrophy in smuts such as Pep1 or important virulence factors such as Srt1 (Hemetsberger et al. 2015; Kijpornyongpan et al. 2018; Lanver et al. 2018). The absence of other such genes in *Tilletia* species correlates with the fact that *Tilletiales* are unique within Exobasidiomycetes and Ustilaginomycotina in several aspects of the life cycle and the ultrastructure of the host-parasite interaction.

#### Secondary metabolites pathways and trimethylamine synthesis

Putative secondary metabolite gene clusters were predicted for *T. caries*, *T. controversa*, and *T. laevis* in this study. Secondary metabolites are known as virulence factors (Oide et al. 2006), toxins, inhibitors (Shwab and Keller 2008), and antifeedants or deterrents (Tanaka et al. 2005; Xu et al. 2019). Non-ribosomal peptide synthetase gene clusters are often repetitive in their internal structures and the identification of a higher number of them in the PacBio sequenced genome can be explained by a higher coverage of repetitive regions. The in silico analyses of putative secondary metabolites gene clusters revealed that they were nearly identical across the three species.

Production of TMA gave the stinking smut its name. The biological function of TMA in *Tilletia* spp. (Ettel and Halbsguth 1963; Singh and Trione 1969) as well as in *Geotrichum candidum* (Robinson et al. 1989) is the autoinhibition of spore germination. Other metabolites of *Tilletia* were shown to inhibit spore germination in vitro (Trione and Ross 1988), but they are not volatile and therefore cannot fulfill the function of autoinhibitors. The precursor of TMA in smut fungi and bacteria is choline, but the lack of homologous genes in smut fungi indicates that the biosynthetic pathway is different. The biosynthesis of TMA in smut fungi also appears unrelated to ethylamine degradation by bacteria. We therefore hypothesize that smut fungi possess a different biosynthetic pathway to TMA, which has yet to be discovered.

### Inter- and intraspecies genomic comparisons

#### Genomic synteny, genome-wide diversity, and phylogenetic inferences

The genomes of the three *Tilletia* species appeared to be largely syntenic as up to 94% of non-repetitive DNA regions could be aligned in a pairwise manner. Furthermore, we detected more than 98.7% average nucleotide identity in one-to-one aligned DNA regions, which was in general agreement with Nguyen et al. (2019) and Sedaghatjoo et al. (2021). The high genomic synteny observed between the three species can be explained by their close phylogenetic relationship (Carris et al. 2007). High genomic identity among closely related species has also been reported for some *Fusarium* species (De Vos et al. 2014) and within *Dothideomycetes* (Ohm et al. 2012).

Genome-wide average diversity was least between *T. caries* and *T. laevis* with 0.51 SNPs/kb and 1.04 indels bp/kb. This observation is in concordance with the results of the phylogenetic analysis based on 241 genes, where representatives of *T. caries* and *T. laevis* did not cluster according to their species affiliation but formed a joint monophyletic group. Independent support for these observations was also found in a study establishing the use of MALDI-TOF MS for differentiation of the three species of interest (Forster et al. 2022). Also in that study, using a broader sample collection, *T. caries* and *T. laevis* could not be resolved as distinct entities. Altogether, this may suggest that the two species could in fact only represent two morphotypes of one species, a so-called pseudomorphospecies (Vánky 2008). It is interesting to note that *T. laevis* is the only species in the genus lacking teliospore ornamentation. However, Vánky (2012) reports that isotypes of *T. laevis* he studied microscopically contained not only smooth teliospores, but also reticulate spores resembling *T. caries* within the same sorus and therefore speculated about a hybridogen origin of the species. In fact, experimental approaches on hybrids of *T. caries* and *T. laevis* point into a similar direction: the hybrid progenies of the crosses between *T. caries* and *T. laevis* displayed teliospores surfaces varying from completely smooth to various degrees of reticulated (Holton 1942). Although the genetic basis for the teliospores reticulation in *Tilletia* species is unknown, in some species of the smut genus *Ustilago* it has been suggested that only two dominant and complementary genes encode for the teliospore wall ornamentation (Nielsen 1968; Huang and Nielsen 1984). If this is also true for *Tilletia* bunts, the different teliospore ornamentation between *T. caries* and *T. laevis* would be caused by different alleles resulting from mutation, because there is no evidence that one phenotype has advantages over the other. This would explain, that overall the genomes can be nearly identical, while a few genes would account for the significant morphological features defining the two species in its current circumscription. Whether this is sufficient to maintain the two species as separate entities – given their basically identical (infection) biology and genome similarity will need to be discussed based on broader specimens samplings in the near future.

At the same time*, T. caries* and *T. laevis* showed almost equal distance to *T. controversa* correlating with the fact that both are identical in germination requirements and infection biology, but different in these from *T. controversa*. This genomic similarity and distances are especially striking, because *T. laevis*, with smooth teliospores, can be relatively easily identified among these three species. In contrast, both *T. caries* and *T. controversa* have ornamented spores differing only slightly and can be problematic to be differentiated morphologically as is required e.g. in seed health testing. However, these two species can be easier distinguished using germination tests requiring different germination time and temperatures, while *T. caries* and *T. laevis* do not differ in this respect.

At species level, up to three times higher nucleotide polymorphisms were observed among the seven isolates of *T. controversa* (max. 1.47 SNPs/kb) compared to the five isolates of *T. caries* (max. 0.53 SNPs/kb) and four isolates of *T. laevis* (max. 0.52 SNPs/kb). This is especially remarkable because the common bunts isolates’ geographic origins were more distant to each other (Austria, Italy, Germany, Switzerland, and USA) compared to those of the dwarf bunt isolates, which were mainly collected from Germany. All in all, the number of SNPs were generally low for all three bunt species compared to reports for other species of Basidiomycota suggesting that the causal agents of common bunt and dwarf bunt might have only diverged recently. For instance, different genotypes of *Heterobasidion irregulare* had 4 SNPs/kb (Sillo et al. 2015) and *Melamspora larici-populina* 6 SNPs/kb (Persoons et al. 2014), respectively. The especially low genetic diversity observed within the common bunt species compared to the slightly higher genetic diversity of *T. controversa*, could be the consequence of different mating systems. All causal agents of common and dwarf bunt display bipolar mating behaviors meaning that selfing by direct mating of compatible basidiospores while still attached to the basidium is the dominant reproduction form (Goates 1996) and is thus limiting the chances of outcrossing. However, the mating system is biallelic in *T. caries* and *T. laevis* (Holton 1951; Holton and Kendrick 1957), while it is multiallelic in *T. controversa* (Hoffmann and Kendrick 1969) leading to a somewhat higher chance of occasional outcrossing and consequently a higher degree of diversity in *T. controversa* as suggested by Pimentel et al. 2000.

#### Inter- and intraspecies diversification

We compared all the predicted protein-coding genes of the three species by clustering orthologues to define variation across the 16 studied isolates and to find genes, which may be involved in different biological features of the three species. The shared fraction of genes between the three species of *T. caries*, *T. controversa*, and *T. laevis* together was 75.4% of the total predicted proteomes. McCarthy and Fitzpatrick (2019) reported that at species level 83.2% and 85% of the total genes represented the core genomes of *Aspergillus fumigatus* and *Saccharomyces cerevisiae*, respectively. Similarly, the size of the core genome of six *Aspergillus niger* isolates was also 80% of the pan-genome (Vesth et al. 2018) while they mostly have asexual reproduction form. The three bunt species together had only 5% lower than the minimum core genome size reported for single fungal species. The high degree of gene conservation between the three *Tilletia* species is in line with the phylogenetic analyses suggesting that *T. caries*, *T. controversa*, and *T. laevis* share a recent common ancestor (Carris et al. 2007).

We surprisingly found very few species-specific genes in our study and their functions could mostly not be predicted in silico. Wet lab analysis is required to determine whether these few genes are involved in the different physiological and morphological features of the three species. It is however noteworthy that the species-specific orthology clusters found were higher in *T. controversa* (21) compared to either *T. caries* (1) or *T. laevis* (3). But if *T. caries* and *T. laevis* were treated as if they were one specific entity, 19 specific orthology clusters could be found corresponding very well with the numbers of specific orthology clusters in *T. controversa* suggesting that they might be hierarchically comparable genetic entities.

## Conclusions

In conclusion, the genomic analyses presented in the present paper show a broad overall similarity in the genomic structure and features of the studied wheat bunt fungi. However, several lines of evidence obtained by the genomic analyses including phylogenetics suggest that the low amount of genomic differences that could reliably be observed reveals a pattern that correlates better with the definition of the diseases ‘common bunt’ versus ‘dwarf bunt’ instead of the three currently defined species.

## Supplementary Information

**Additional file 1: Table 1.** List of the genome assemblies, raw reads, and RNA-Seq data used in this study. **Table 2.** List of the additional Basidiomycetes genomes and their accession numbers used for gene model prediction. **Table 3.** Assessment of the genome annotation completeness when different approaches (homology-based, ab initio and a combination) were used. **Table 4.** Percentage of repetitive regions in five isolates of *T. caries*, seven isolates of *T. controversa,* and four isolates of *T. laevis*. **Table 5.** Number and relative abundance of total SSRs identified in the *T. caries*, *T. controversa*, and *T. laevis* genomes. **Table 6.** Percentage of the maximum aligned bases in non-repetitive regions. **Table 7.** The number of putative enzymes in each CAZyme family in different isolates and their substrate. **Table 8.** Prediction of the secondary metabolite biosynthesis gene clusters. **Table 9.** Analysis of adenylation domains of putative NRPS genes in *Tilletia* spp. **Table 10.** List of the identified orthology clusters. **Table 11.** In silico functional prediction of the species-specific orthology clusters.

**Additional file 2: Figure 1.** Coverage of putative secondary metabolites and biosynthetic gene clusters. Trimmed Illumina reads of each isolate were aligned to the selected reference gene cluster sequence using default mapping parameters. The plot shows the read depth up to 500 (blue line) and lowest regression (filter: 1/25; orange line). The median coverage is indicated on the right y-axis in red and the x-axes shows length of the predicted secondary metabolite gene cluster in bp. Regions which were annotated as core genes by antiSMASH are shaded in grey. The reference secondary metabolite gene clusters used for mappings are as follow: The predicted gene cluster of *T. controversa* strain OR for, Indole, NRPS-like I, NRPS-like II, T1PKS/NRPS-like, *T. caries* isolate AA11 for NRPS, Terpene I, Terpene II, *T. laevis* isolate ATCC 42080 for NRPS-like IV, and *T. controversa* isolate DAOMC 236426 for NRPS-like III. The region marked with arrow represent non-covered regions by reads. While the present and the order of nearly all gene clusters were also supported by Illumina reads, the observed drop in reads coverage in T1PKS/NRPS-like synthases was probably due to the present of long stretches of Ts and Ns nucleotides within this gene cluster in our selected reference. Part of the NRPS-like I gene cluster in the *T. caries* isolate DOAMC 238032 could not be recovered by mapping.

## Data Availability

The datasets generated during the current study (raw reads, assemblies, and structural annotations) are available in the European Nucleotide Archive database repository (http://www.ebi.ac.uk/ena) and can be accessed under the project accession number of PRJEB40624.

## Abbreviations

AA: Auxiliary Activity
CAZymes: Carbohydrate-active enzymes
CBM: Carbohydrate-Binding Module
CDS: Coding sequences
CE: Carbohydrate Esterase
GH: Glycoside Hydrolase
GT: Glycosyltransferases
indel: Small insertions and deletion
LTR: Long terminal direct repeats
NRPS: Non-ribosomal peptide like synthases
PCWDE: Plant cell wall-degrading enzyme
SNP: Single nucleotide polymorphism
SSR: Simple sequence repeat
PL: Polysaccharide Lyases
TE: Transposable element
TMA: Trimethylamine
tRNA: Transfer RNA
T1PKS: Type 1 polyketide synthases
