# Supplementary material for: Comparative genomics reveals low levels of inter- and intraspecies diversity in the causal agents of dwarf and common bunt of wheat and hint at conspecificity of Tilletia caries and T. laevis
Source: IMA Fungus. 2022 Jun 7;13:11. doi: 10.1186/s43008-022-00098-y (PMC9172201; doi:10.1186/s43008-022-00098-y)
Supplement: Supplementary file 2 — Additional file 2: Figure 1. Coverage of putative secondary metabolites and biosynthetic gene clusters. Trimmed Illumina reads of each isolate were aligned to the selected reference gene cluster sequence using default mapping parameters. The plot shows the read depth up to 500 (blue line) and lowest regression (filter: 1/25; orange line). The median coverage is indicated on the right y-axis in red and the x-axes shows length of the predicted secondary metabolite gene cluster in bp. Regions which were annotated as core genes by antiSMASH are shaded in grey. The reference secondary metabolite gene clusters used for mappings are as follow: The predicted gene cluster of T. controversa strain OR for, Indole, NRPS-like I, NRPS-like II, T1PKS/NRPS-like, T. caries isolate AA11 for NRPS, Terpene I, Terpene II, T. laevis isolate ATCC 42080 for NRPS-like IV, and T. controversa isolate DAOMC 236426 for NRPS-like III. The region marked with arrow represent non-covered regions by reads. While the present and the order of nearly all gene clusters were also supported by Illumina reads, the observed drop in reads coverage in T1PKS/NRPS-like synthases was probably due to the present of long stretches of Ts and Ns nucleotides within this gene cluster in our selected reference. Part of the NRPS-like I gene cluster in the T. caries isolate DOAMC 238032 could not be recovered by mapping. [file 43008_2022_98_MOESM2_ESM.pdf]

Comparative genomics reveals low levels of inter- and intraspecies diversity in the causal agents of dwarf and common bunt of wheat and hint at conspecificity of *Tilletia caries* and *T. laevis*

Somayyeh Sedaghatjoo[1], Bagdevi Mishra[2], Monika K. Forster[3], Yvonne Becker[1], Jens Keilwagen[4], Berta Killermann[3], Marco Thines[5], Petr Karlovsky[6*], Wolfgang Maier[1*]

Email of the corresponding author: s.sedaghatjoo@gmail.com

[1] Julius Kühn Institute (JKI) – Federal Research Centre for Cultivated Plants, Institute for Epidemiology and Pathogen Diagnostics, Messeweg 11-12, 38104 Braunschweig, Germany

[2] Senckenberg Biodiversity and Climate Research Centre, Senckenberganlage 25, 60325 Frankfurt am Main, Germany

[3] Bavarian State Research Center for Agriculture, Institute for Crop Science and Plant Breeding, Vöttinger Straße 38, 85354 Freising, Germany

[4] Julius Kühn Institute (JKI) – Federal Research Centre for Cultivated Plants, Institute for Biosafety in Plant Biotechnology, Erwin-Baur-Str. 27, 06484 Quedlinburg, Germany

[5] Goethe University, Faculty of Biological Sciences, Institute of Ecology, Evolution and Diversity, Max-von-Laue-Str. 13, 60438 Frankfurt am Main, Germany

[6] Molecular Phytopathology and Mycotoxin Research, University of Goettingen, Grisebachstrasse 6, 37077 Goettingen, Germany

*Joint senior authors, listed in alphabetical order.

Indole

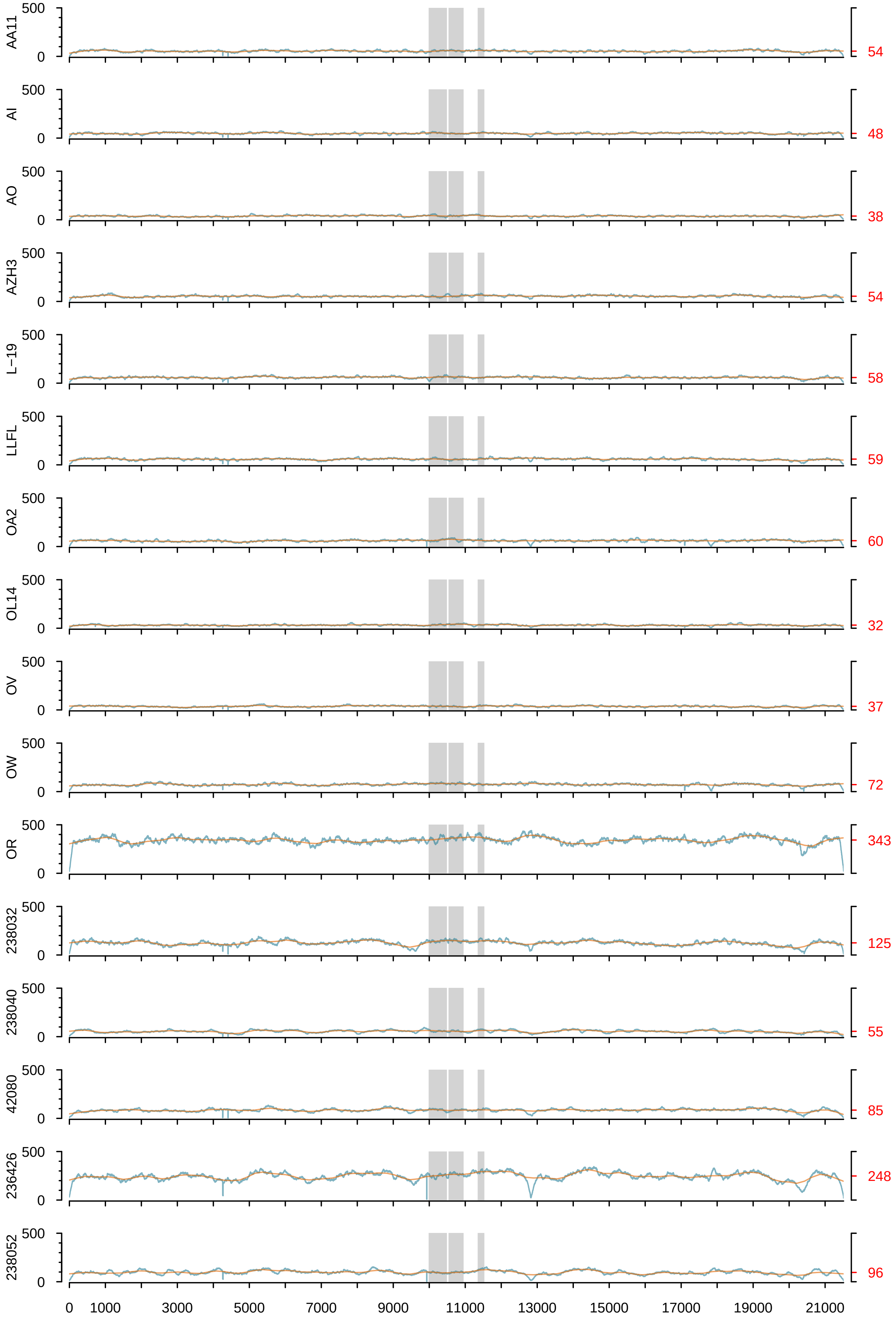

AA11
AI
AO
AZH3
L−19
LLFL
OA2
OL14
OV
OW
OR
238032
238040
42080
236426
238052
500
0
54
48
38
54
58
59
60
32
37
72
343
125
55
85
248
96
0
1000
3000
5000
7000
9000
11000
13000
15000
17000
19000
21000

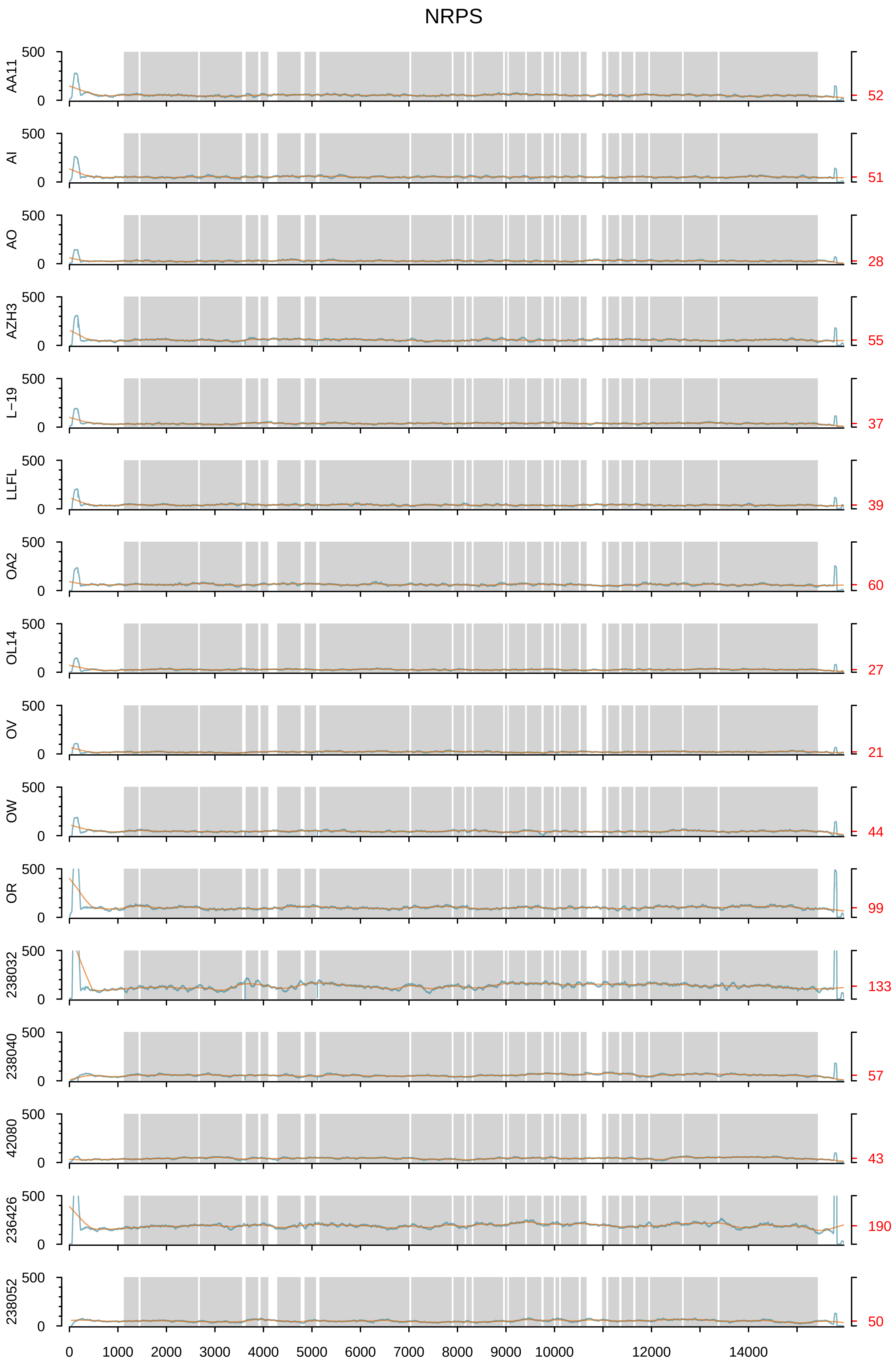

NRPS
AA11
AI
AO
AZH3
L−19
LLFL
OA2
OL14
OV
OW
OR
238032
238040
42080
236426
238052
500
0
52
51
28
55
37
39
60
27
21
44
99
133
57
43
190
50
0
1000
2000
3000
4000
5000
6000
7000
8000
9000
10000
12000
14000

NRPS−like I

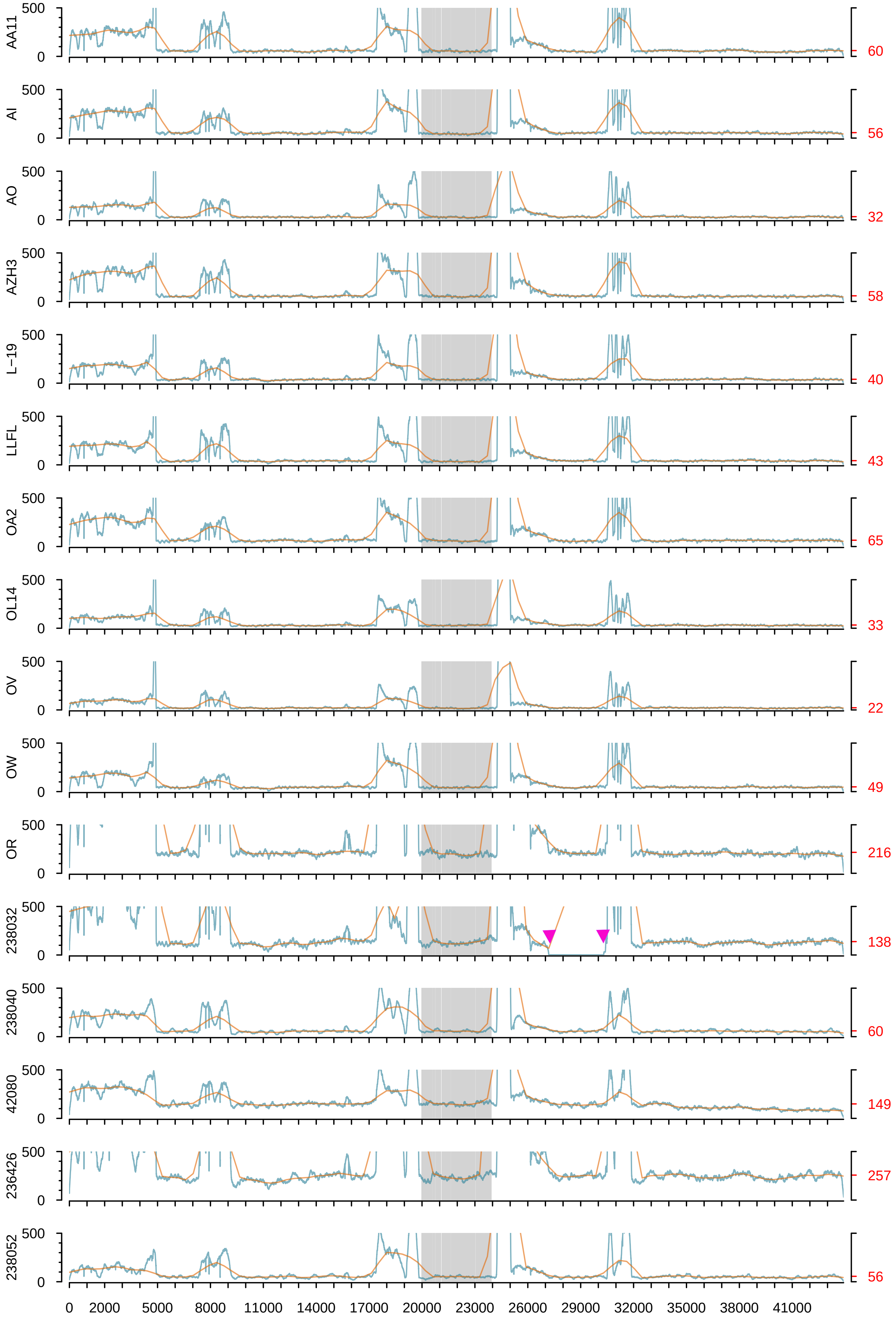
AA11
AI
AO
AZH3
L−19
LLFL
OA2
OL14
OV
OW
OR
238032
238040
42080
236426
238052
500
0
60
56
32
58
40
43
65
33
22
49
216
138
60
149
257
56
0 2000 5000 8000 11000 14000 17000 20000 23000 26000 29000 32000 35000 38000 41000

NRPS−like II

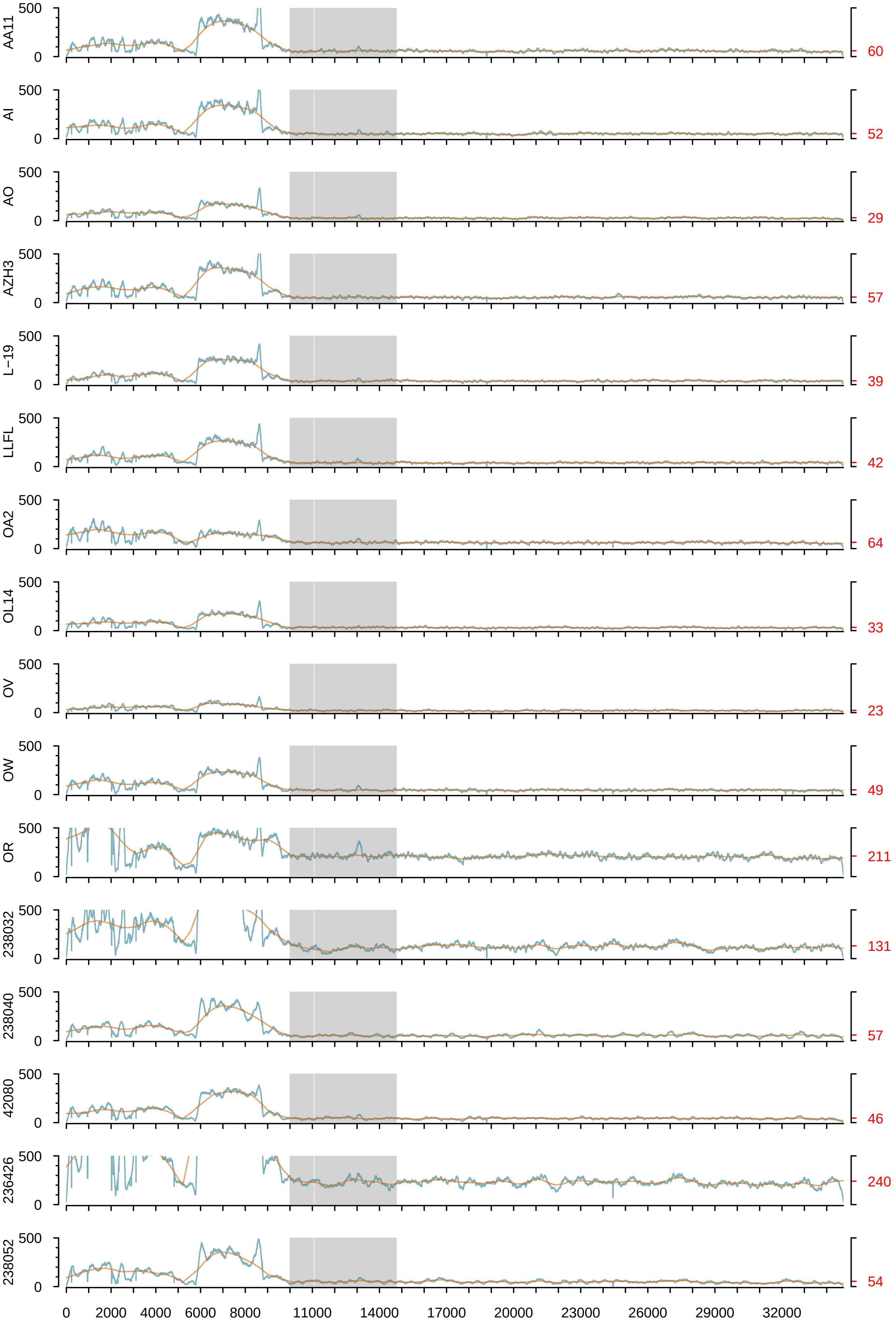
AA11
AI
AO
AZH3
L−19
LLFL
OA2
OL14
OV
OW
OR
238032
238040
42080
236426
238052
60
52
29
57
39
42
64
33
23
49
211
131
57
46
240
54
0
500
0
2000
4000
6000
8000
11000
14000
17000
20000
23000
26000
29000
32000

NRPS−like III

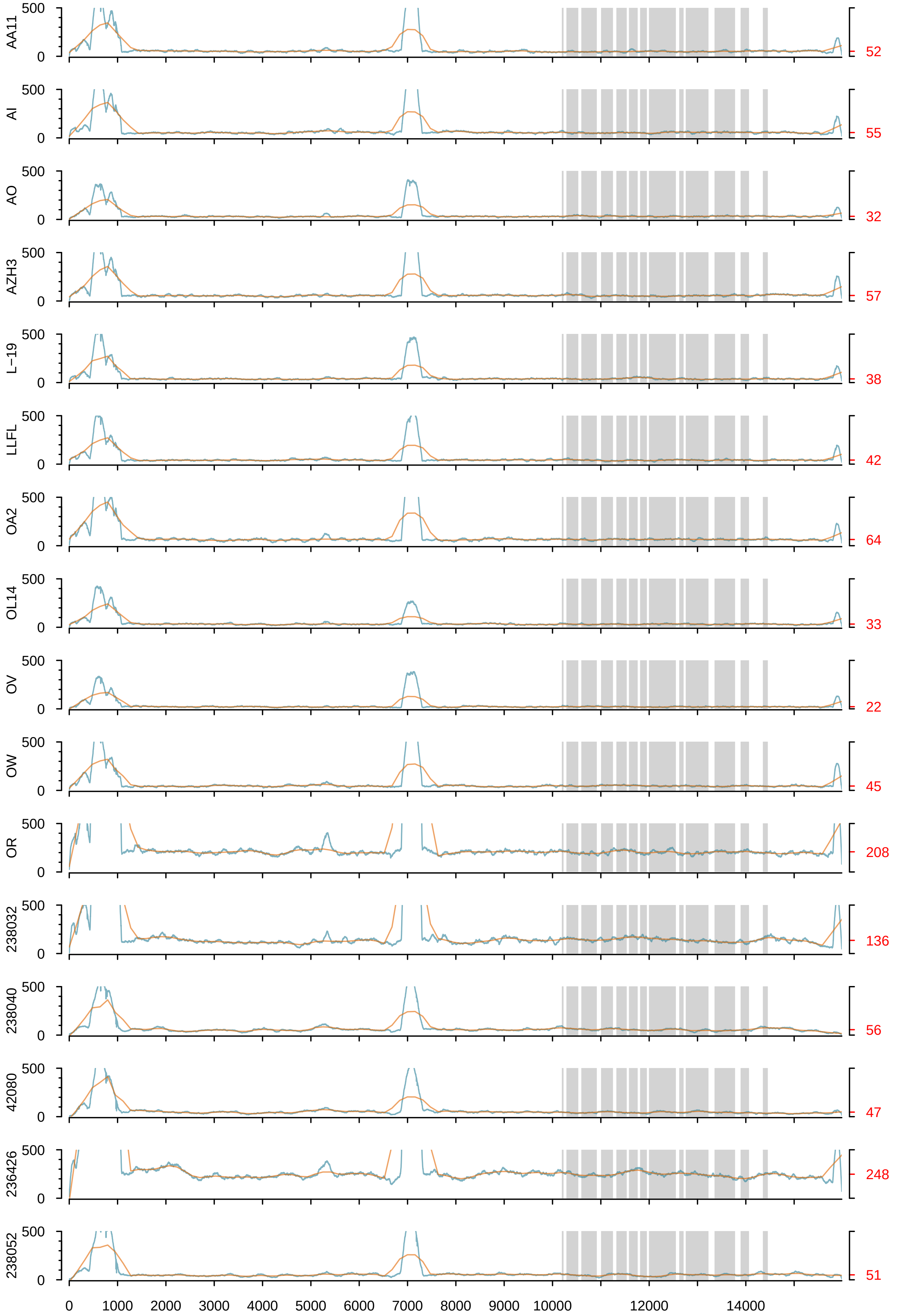

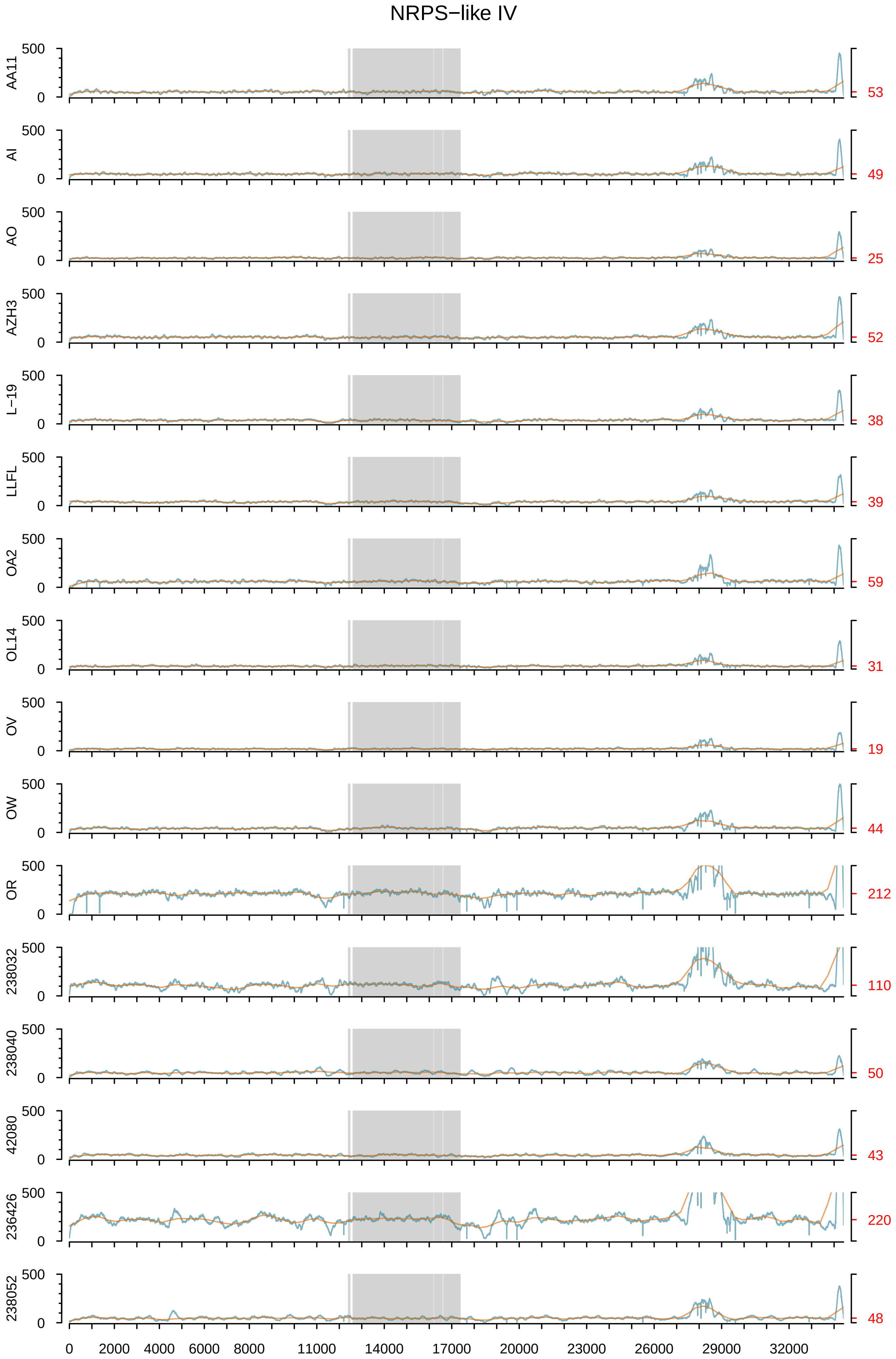

NRPS−like IV
AA11
AI
AO
AZH3
L−19
LLFL
OA2
OL14
OV
OW
OR
238032
238040
42080
236426
238052
500
0
53
49
25
52
38
39
59
31
19
44
212
110
50
43
220
48
0
2000
4000
6000
8000
11000
14000
17000
20000
23000
26000
29000
32000

Terpene I

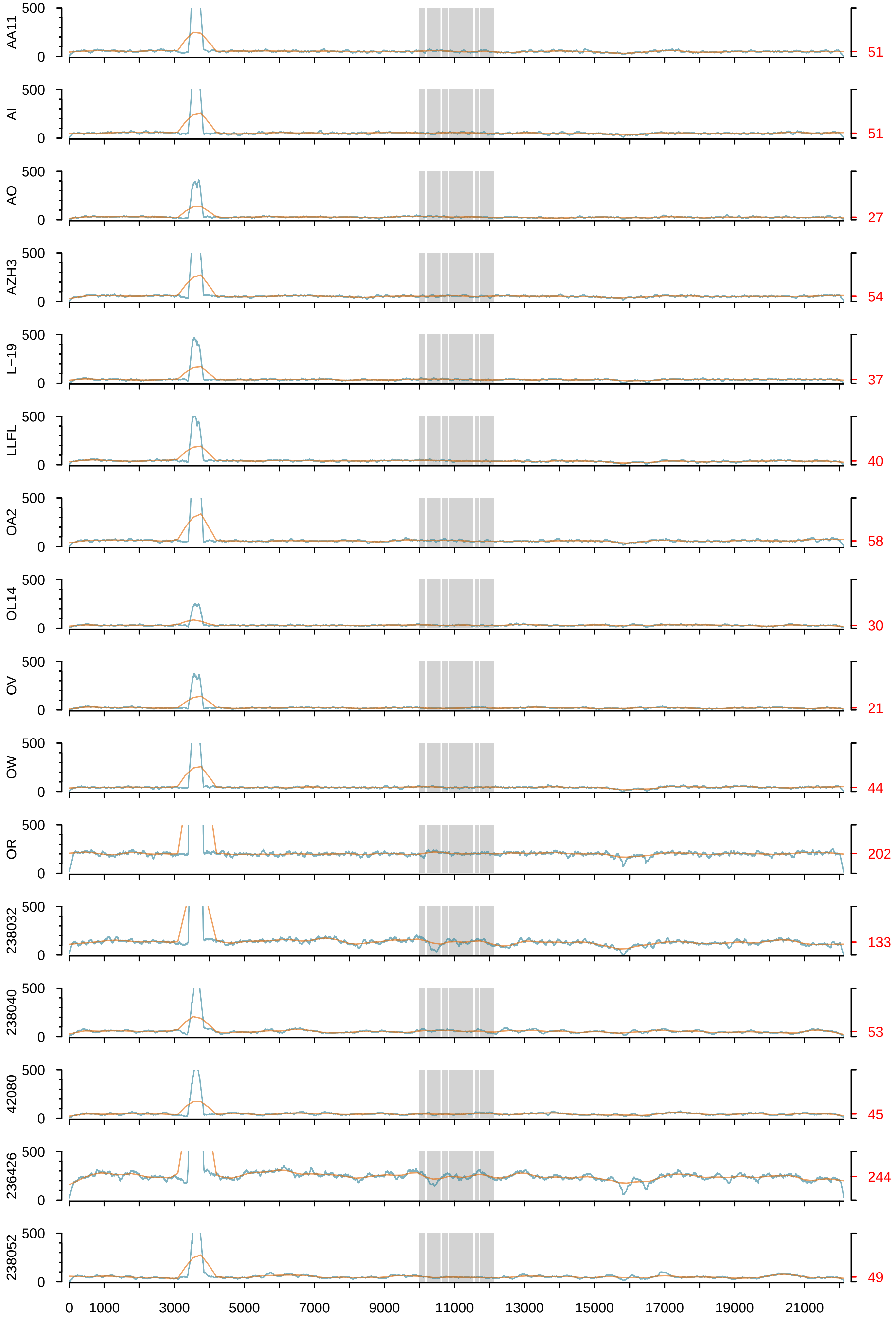
AA11
AI
AO
AZH3
L−19
LLFL
OA2
OL14
OV
OW
OR
238032
238040
42080
236426
238052
500
0
51
51
27
54
37
40
58
30
21
44
202
133
53
45
244
49
0
1000
3000
5000
7000
9000
11000
13000
15000
17000
19000
21000

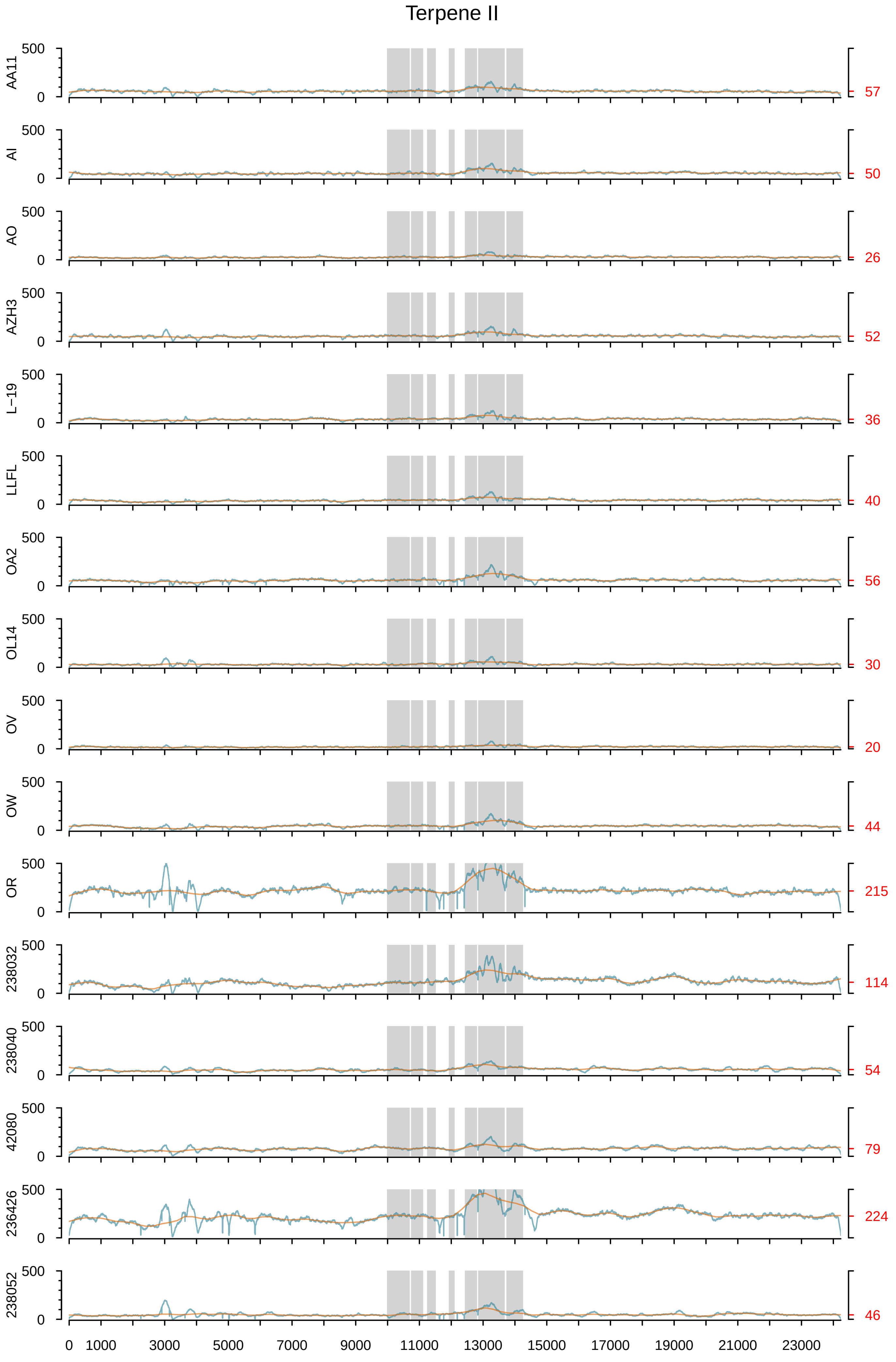

Terpene II
AA11
AI
AO
AZH3
L−19
LLFL
OA2
OL14
OV
OW
OR
238032
238040
42080
236426
238052
500
0
57
50
26
52
36
40
56
30
20
44
215
114
54
79
224
46
0
1000
3000
5000
7000
9000
11000
13000
15000
17000
19000
21000
23000

T1PKS/NRPS−like

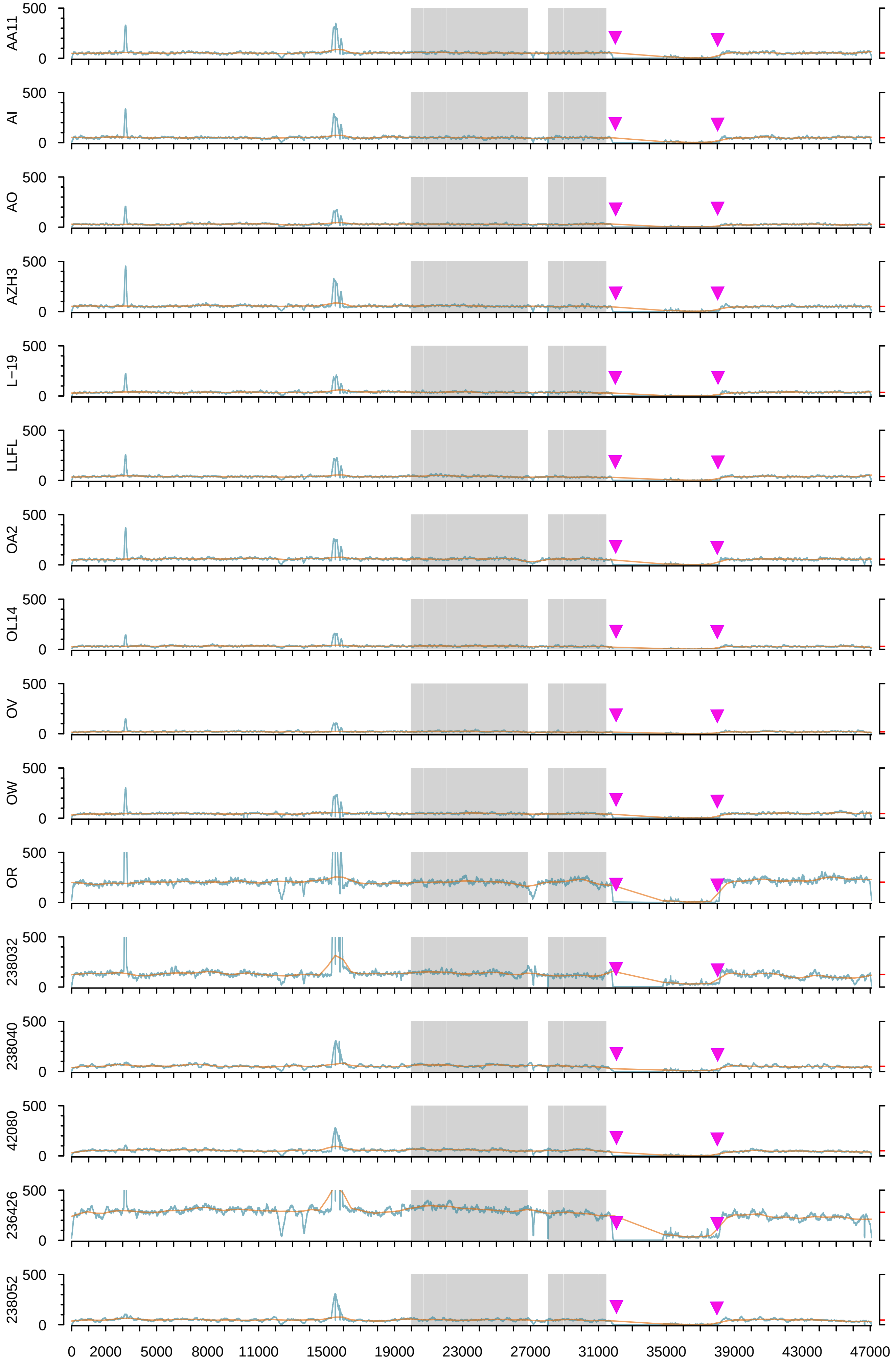

Additional Figure 1 Coverage of putative secondary metabolites and biosynthetic gene clusters. Trimmed Illumina reads of each isolate were aligned to the selected reference gene cluster sequence using default mapping parameters. The plot shows the read depth up to 500 (blue line) and the lowest regression (filter: 1/25; orange line). The median coverage is indicated on the right y-axis in red and the x-axes shows length of the predicted secondary metabolite gene cluster in bp. Regions which were annotated as core genes by antiSMASH are shaded in gray. The reference secondary metabolite gene clusters used for mappings are as follow: The predicted gene cluster of *T. controversa* strain OR for, Indole, NRPS-like I, NRPS-like II, T1PKS/NRPS-like, *T. caries* isolate AA11 for NRPS, Terpene I, Terpene II, *T. laevis* isolate ATCC 42080 for NRPS-like IV, and *T. controversa* isolate DAOMC 236426 for NRPS-like III. The region marked with arrow represent non-covered regions by reads. While the present and the order of nearly all gene clusters were also supported by Illumina reads, the observed drop in reads coverage in T1PKS/NRPS-like synthases was probably due to the present of long stretches of Ts and Ns nucleotides within this gene cluster in our selected reference. Part of the NRPS-like I gene cluster in the *T. caries* isolate DOAMC 238032 could not be recovered by mapping.
